# A New Natural Language Processing–Inspired Methodology (Detection, Initial Characterization, and Semantic Characterization) to Investigate Temporal Shifts (Drifts) in Health Care Data: Quantitative Study

**DOI:** 10.2196/54246

**Published:** 2024-10-28

**Authors:** Bruno Paiva, Marcos André Gonçalves, Leonardo Chaves Dutra da Rocha, Milena Soriano Marcolino, Fernanda Cristina Barbosa Lana, Maira Viana Rego Souza-Silva, Jussara M Almeida, Polianna Delfino Pereira, Claudio Moisés Valiense de Andrade, Angélica Gomides dos Reis Gomes, Maria Angélica Pires Ferreira, Frederico Bartolazzi, Manuela Furtado Sacioto, Ana Paula Boscato, Milton Henriques Guimarães-Júnior, Priscilla Pereira dos Reis, Felício Roberto Costa, Alzira de Oliveira Jorge, Laryssa Reis Coelho, Marcelo Carneiro, Thaís Lorenna Souza Sales, Silvia Ferreira Araújo, Daniel Vitório Silveira, Karen Brasil Ruschel, Fernanda Caldeira Veloso Santos, Evelin Paola de Almeida Cenci, Luanna Silva Monteiro Menezes, Fernando Anschau, Maria Aparecida Camargos Bicalho, Euler Roberto Fernandes Manenti, Renan Goulart Finger, Daniela Ponce, Filipe Carrilho de Aguiar, Luiza Margoto Marques, Luís César de Castro, Giovanna Grünewald Vietta, Mariana Frizzo de Godoy, Mariana do Nascimento Vilaça, Vivian Costa Morais

**Affiliations:** 1 Computer Science Department, Universidade Federal de Minas Gerais, Belo Horizonte, Brazil Belo Horizonte Brazil; 2 Computer Science Department, Universidade Federal de São João del-Rei, Brazil São João del-Rei Brazil; 3 Faculdade de Medicina, Universidade Federal de Minas Gerais, Belo Horizonte, Brazil Belo Horizonte Brazil; 4 Hospitais da Rede Mater Dei Belo Horizonte Brazil; 5 Hospital de Clínicas de Porto Alegre Porto Alegre Brazil; 6 Hospital Santo Antônio Curvelo Brazil; 7 Faculdade Ciências Médicas de Minas Gerais Belo Horizonte Brazil; 8 Hospital Tacchini Bento Gonçalves Brazil; 9 Hospital Márcio Cunha Ipatinga Brazil; 10 Hospital Metropolitano Doutor Célio de Castro Belo Horizonte Brazil; 11 Hospital Risoleta Tolentino Neves Belo Horizonte Brazil; 12 Faculdade de Medicina, Universidade Federal dos Vales do Jequitinhonha e Mucuri Teófilo Otoni Brazil; 13 Hospital Santa Cruz Santa Cruz do Sul Brazil; 14 Hospital Semper Belo Horizonte Brazil; 15 Hospital Unimed BH Belo Horizonte Brazil; 16 Hospital Universitário de Santa Maria Santa Maria Brazil; 17 Hospital Moinhos de Vento Porto Alegre Brazil; 18 Hospital Nossa Senhora da Conceição Porto Alegre Brazil; 19 Fundação Hospitalar do Estado de Minas Gerais Belo Horizonte Brazil; 20 Hospital Mãe de Deus Porto Alegre Brazil; 21 Hospital Regional do Oeste Chapecó Brazil; 22 Faculdade de Medicina de Botucatu, Universidade Estadual Paulista Júlio de Mesquita Filho Botucatu Brazil; 23 Hospital das Clínicas, Universidade Federal de Pernambuco Recife Brazil; 24 Hospital Bruno Born Lajeado Brazil; 25 Hospital SOS Cárdio Florianópolis Brazil; 26 Hospital Metropolitano Odilon Behrens Belo Horizonte Brazil

**Keywords:** health care, machine learning, data drifts, temporal drifts

## Abstract

**Background:**

Proper analysis and interpretation of health care data can significantly improve patient outcomes by enhancing services and revealing the impacts of new technologies and treatments. Understanding the substantial impact of temporal shifts in these data is crucial. For example, COVID-19 vaccination initially lowered the mean age of at-risk patients and later changed the characteristics of those who died. This highlights the importance of understanding these shifts for assessing factors that affect patient outcomes.

**Objective:**

This study aims to propose detection, initial characterization, and semantic characterization (DIS), a new methodology for analyzing changes in health outcomes and variables over time while discovering contextual changes for outcomes in large volumes of data.

**Methods:**

The DIS methodology involves 3 steps: detection, initial characterization, and semantic characterization. Detection uses metrics such as Jensen-Shannon divergence to identify significant data drifts. Initial characterization offers a global analysis of changes in data distribution and predictive feature significance over time. Semantic characterization uses natural language processing–inspired techniques to understand the local context of these changes, helping identify factors driving changes in patient outcomes. By integrating the outcomes from these 3 steps, our results can identify specific factors (eg, interventions and modifications in health care practices) that drive changes in patient outcomes. DIS was applied to the Brazilian COVID-19 Registry and the Medical Information Mart for Intensive Care, version IV (MIMIC-IV) data sets.

**Results:**

Our approach allowed us to (1) identify drifts effectively, especially using metrics such as the Jensen-Shannon divergence, and (2) uncover reasons for the decline in overall mortality in both the COVID-19 and MIMIC-IV data sets, as well as changes in the cooccurrence between different diseases and this particular outcome. Factors such as vaccination during the COVID-19 pandemic and reduced iatrogenic events and cancer-related deaths in MIMIC-IV were highlighted. The methodology also pinpointed shifts in patient demographics and disease patterns, providing insights into the evolving health care landscape during the study period.

**Conclusions:**

We developed a novel methodology combining machine learning and natural language processing techniques to detect, characterize, and understand temporal shifts in health care data. This understanding can enhance predictive algorithms, improve patient outcomes, and optimize health care resource allocation, ultimately improving the effectiveness of machine learning predictive algorithms applied to health care data. Our methodology can be applied to a variety of scenarios beyond those discussed in this paper.

## Introduction

### Overview

Health care data are a critical resource that can be used to improve patient outcomes and the financial performance of health care institutions [1,2]. By analyzing patient data, health care providers can gain insights into patients’ health status, identify trends, and make informed decisions about treatment plans. Properly collected, managed, treated, and interpreted health care data can help providers improve operational efficiency and reduce costs, thereby improving financial results [3].

One of the primary ways health care data can be used to enhance medical decisions and potentially improve patient outcomes is through predictive analysis. This technique uses historical data to identify patterns and predict future outcomes, thereby enabling the recognition of high-risk patients, the simulation of different therapeutic approaches, and the personalization of patient care. However, relying on historical data has its caveats, as the predictive capacity of different variables is not fixed over time. Ignoring these aspects of temporal data may lead to prediction errors and learning instabilities. These variations in performance are part of what is known as temporal data shifts [4-7].

A temporal data shift refers to a change in the statistical properties of a data set over time, which can degrade model accuracy. In health care, this may occur due to various reasons, including changes in data collection practices, software updates or replacements, changes in patient behavior or lifestyle habits, and the introduction of new therapeutic technologies. These temporal events may lead to inconsistencies and discrepancies in the data, which may affect both the accuracy and reliability of the data and models trained on them. The impacts can be significant [4,7], as they can lead to incorrect diagnoses, inappropriate treatment plans, and poor patient outcome predictions. This highlights the importance of managing, characterizing, and mitigating these temporal effects [8].

We are particularly interested in how temporal data drifts can be used to analyze the effectiveness of new patient treatment options. Changes in predictive capacity can provide insights into the impact of new treatments on patient outcomes. For instance, by comparing data collected before and after introducing a new treatment, we can identify any shifts that may indicate improved patient outcomes. If the data drift analysis indicates a positive impact of the new treatment, health care providers may choose to continue to monitor the data to ensure that the positive effects are sustained while maintaining the use of the new therapeutic option [9].

A notable example of a condition that experienced an important data drift over time is the HIV infection. In the 1980s, HIV infection was a strong predictor of early death. However, it has now become more of a chronic condition, such as diabetes mellitus or systemic hypertension. In the same manner, advancements in breast cancer treatment have significantly increased survivorship over the years [10].

Similarly, several infectious diseases, such as poliomyelitis or measles, have been nearly eradicated in most parts of the world, making them unlikely hypotheses for new diagnoses [11,12]. In the case of COVID-19, vaccination has dramatically changed the profile of hospitalizations and deaths [4,13], initially decreasing the mean age of patients at risk and creating a clear distinction between the periods before and after vaccination.

### Our Main Contribution: The Detection, Initial Characterization, and Semantic Characterization Methodology

**Figure 1:**
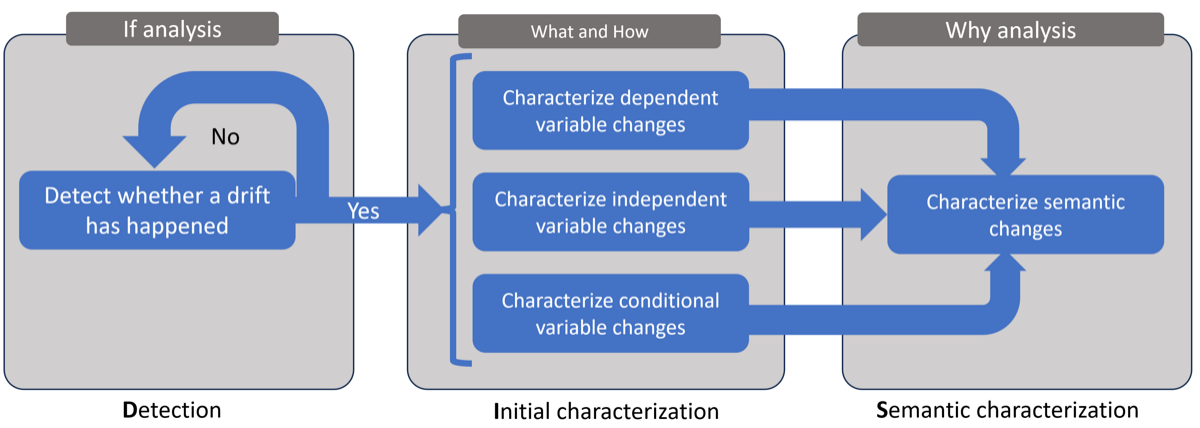
Overview of the detection, initial characterization, and semantic characterization (DIS) methodology.

Building upon the idea of analyzing data drifts to obtain insights into how and whether new technologies or treatments have impacted patient outcomes, this paper proposes a novel, 3-step health care temporal analysis methodology, called detection, initial characterization, and semantic characterization (DIS). The proposed DIS methodology is summarized in Figure 1. It consists of three main steps, (1) detection, (2) initial characterization, and (3) semantic characterization, which are described in the following sections.

In summary, we exploited various drift detection metrics in the detection step to identify any significant instances of data drift. Some of the metrics we explored in this step include Jensen-Shannon divergence [14], autoencoder reconstruction error [15], and centroid distances [16]. If changes were detected, we proceeded to the initial characterization step, where we obtained a global (data set–level) descriptive analysis of what changed and how the discriminative and predictive power of each feature and the distribution of labels evolved over time.

Additionally, we introduced the concept of temporal granularity in the data drift domain, which holds particular significance in health care data drifts and influences the instantiation of our third and final step. High temporal granularity is observed when a data set allows the visualization of numerous events over time for individual patients, with a clear understanding of the chronological order among these events. Conversely, low temporal granularity is observed when each patient is considered a singular event in time, lacking clarity regarding the precedence or sequence of different attributes.

Finally, guided by these principles, we proceeded to the third, semantic characterization step, which exploits concepts popularized in the natural language processing (NLP) domain to provide a localized (instance-level) perspective of why certain shifts occurred. To achieve this, we exploited vector embeddings derived from health care events, such as sequences of the *International Classification of Diseases (ICD)* codes, vital data measurements, and consumption items. Each of these semantic units (*ICD* codes, measurements, consumed items, etc) was treated as an “event” or, in NLP terminology, a “token.” By using NLP-inspired techniques to create semantic embeddings for these entities, we aimed to uncover insights into the changing context and its impact on the outcomes of interest over time.

Before delving into the details of each step in our methodology, it is crucial to emphasize that our DIS approach differs significantly from common practices. While conventional methods usually involve an ad hoc combination of techniques for data collection, qualitative data processing and extraction, and data analysis, our DIS methodology offers a planned and structured procedure, as illustrated in Figure 1. This procedure delineates the required steps to understand data drift in health care data. As we will demonstrate and discuss, these steps can be tailored to various case studies by applying different techniques depending on specific data characteristics. We also offer guidance for selecting one particular approach for a given scenario. Furthermore, we discuss how the results of each step can inform the execution of the following ones and how the combined results of all steps can support our understanding of the drift.

More broadly, to the best of our knowledge, this is the first study to examine data drifts in health care from a technology incorporation standpoint. Rather than solely focusing on enhancing the robustness of machine learning (ML) models, we delved into the underlying factors driving temporal shifts in patient outcomes. Our aim was to study the impact of emerging technologies such as new drugs, patient care policies, or vaccines. In the following sections, we detail the steps of our DIS methodology and illustrate its application in 2 case studies with distinct characteristics in terms of temporal data shifts: the Brazilian COVID-19 Registry data set [17] and the Medical Information Mart for Intensive Care, version IV (MIMIC-IV) data set [18]. By doing so, we illustrate how DIS can obtain insights into the reasons behind some real-life data drifts, as well as their potential impacts, both positive and negative, from a health care perspective.

The main contributions of this paper are summarized in Textbox 1.

**Main contributions of our study.**

**Contributions**

1. The proposal of a new data drift characterization and analysis methodology, detection, initial characterization, and semantic characterization (DIS), that is flexible enough to work on different scenarios. DIS encapsulates and cohesively organizes a sequence of necessary steps for data drift analysis.
2. A new semantic analysis step based on natural language processing embeddings for temporal understanding, which focuses on comprehending the context of relevant outcomes by examining changes in their embedding vectors over time. By incorporating such semantic techniques, DIS provides deeper insights into the reasons behind temporal changes, especially when combined with domain-specific knowledge. This approach allows for a more nuanced analysis of data evolution over time, capturing complex patterns and relationships that may not be apparent with traditional methods such as cluster analysis.
3. The application of the DIS methodology to 2 different case studies with very different temporal granularity profiles illustrates the possibility of conducting insightful analyses using the methodology. We also offer guidelines to aid practitioners in making informed decisions about which methods to use in each step of our methodology, based on particular characteristics of the data. This demonstrates the generalizability and applicability of DIS across different scenarios.

## Methods

### A Detailed Description of the DIS Methodology

#### Detection Step

In step 1 (detection), the main focus is on assessing whether the data have relevant temporal variations. Monitoring and detecting such data drifts are crucial for upholding the accuracy and reliability of ML models and for identifying beneficial and detrimental changes in health care caused by interventions, such as the introduction of new treatments or drugs. From the perspective of a health care service or company, this step identifies whether changes are occurring, potentially prompting further investigations that could enhance service efficiency over time.

For the *detection step*, we recommend splitting the data into temporal chunks and then comparing the data distributions in consecutive chunks. A drift is detected whenever the distributions of distinct chunks exhibit significant differences. Various metrics to compare empirical distributions are available in the literature. These metrics have different characteristics and underlying principles, which may lead to relevant differences in their effectiveness in detecting temporal data drifts. In this work, we considered the following metrics: centroid cosine distance [16], Jensen-Shannon divergence [14], autoencoder reconstruction error [15], classifier error (in separating 2-time chunks) [19], and principal component analysis (PCA) reconstruction error [20] metrics.

The centroid cosine distance metric assesses changes in the central points of data clusters over time and is sensitive to numeric outliers, particularly in heavy-tailed distributions where extremes can be multiple orders of magnitude larger than typical values. The PCA reconstruction error captures variations in data structure by quantifying the difference between original and reconstructed data. Similarly, autoencoder reconstruction error focuses on reconstruction accuracy. Both metrics measure the “novelty” of a data point and are sensitive to numerical outliers. By contrast, the classifier error evaluates a model’s ability to distinguish past from future data, providing insights into how drift affects predictive capabilities. Finally, the Jensen-Shannon divergence quantifies distributional changes, offering a broader perspective on underlying data distribution shifts over time. While reconstruction errors and centroids excel at detecting local outliers and structural changes, the Jensen-Shannon divergence and classifier error provide a more comprehensive view of distributional shifts, making them valuable for modeling the impact of temporal drifts on data distributions.

As an example, our prior analysis of the Brazilian COVID-19 Registry [17] revealed a data drift that significantly impacted the death prediction task, suggesting that vaccination had a pivotal role in the profiles of hospitalized and deceased patients during the COVID-19 pandemic [4]. Although this is an interesting finding, the previous study did not present a proper structure to detect, monitor, and interpret such drifts generically, nor did it propose mechanisms to detect semantic information associated with specific outcomes.

**Figure 2:**
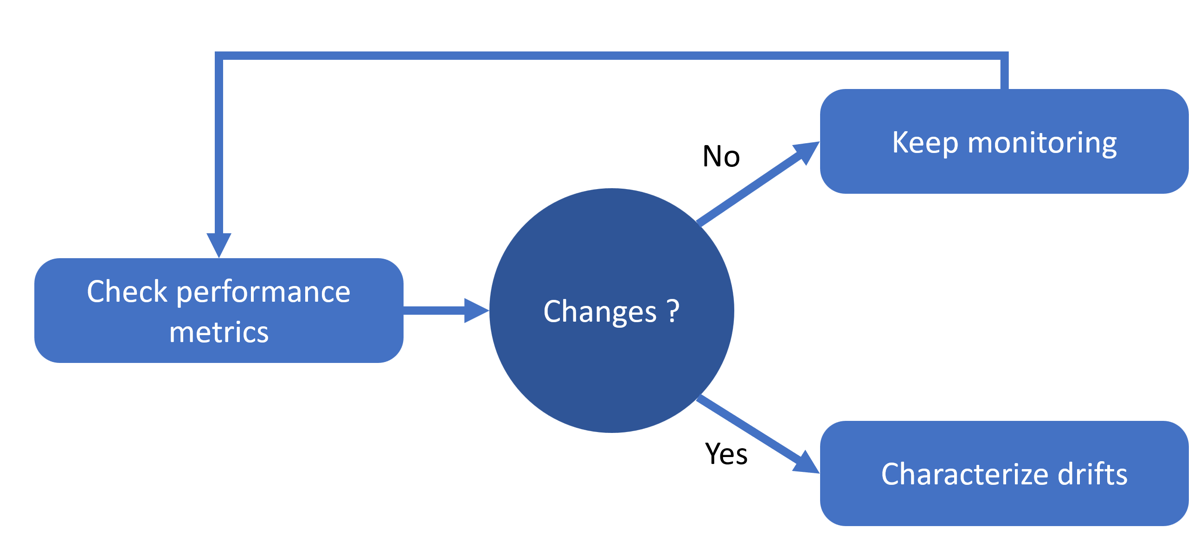
The temporal drift monitoring loop. We usually observe temporal shifts as important variations in model effectiveness over time.

The drift caused by vaccination can be initially hypothesized by comparing the data distributions of consecutive chunks (eg, near future vs recent past) using a classification approach. This involves monitoring the prediction model’s performance over time using metrics such as accuracy, precision, and recall. Alternatively, the distribution of different features over time can be tracked using metrics such as Jensen-Shannon divergence or autoencoder reconstruction errors. If the model’s performance drops (or changes) significantly over time or if the differences between the metrics exceed a certain threshold, it may indicate a data drift. A summary of this monitoring loop is illustrated in Figure 2.

#### Initial Characterization Step

Once a drift has been detected, we proceed to step 2 (initial characterization), where we begin to understand, from a global perspective (all data), *how* the data have changed (Table 1 [21]). This stage focuses on developing a general (global) comprehension of the *whats* and *hows* contributing to the changes observed in the data collection. Specifically, we are interested in characterizing variations in both *dependent P*(*y*) and independent *P*(*x*) variables, as well as the conditional probability of the dependent variables given the independent variables *P*(*y*|*x*). To reach these goals, we examine how *P*(*y*) has changed by plotting its frequency over time; the same is valid for *P*(*x*). For *P*(*y|x*), we can explore different complementary techniques that can help understand the drifts globally. We can analyze how the different correlation metrics between the top independent variables and the dependent variable change over time, for instance, with Pearson [22] or Spearman [23] correlations, or analyze the feature importance of tree-based learners or entropy-based measures such as information gain or chi-square over time [24]. Another possibility is to exploit explainability metrics based on game theory, such as Shapley additive explanations values [25].

“Sudden drift” describes a situation where changes are abrupt and usually caused by a single event, such as a change in data collection practices, where an attribute stops being collected. “Incremental drift” describes gradual and directional changes in a data distribution, such as the observed increase in the populations with overweight and obesity over the past years. “Gradual drift” is similar but does not imply directional changes. Instead, it encompasses other gradual changes, such as the slow change in the hospital admission profile over many years. Finally, “reoccurring drift” refers to a drift pattern that repeats over time, such as the seasonal increase in emergency services admitting patients with influenza during predictable seasons of the year.

This type of analysis facilitates understanding how the relationship between predictive variables and the outcome of interest has evolved from a global perspective. Additionally, it is helpful to check the rate of change for each selected outcome by using similarity metrics and comparing the different groups of patients over time. At this stage, it is feasible to answer valuable research and business questions. For instance, we may observe a decreased likelihood of the “death” outcome in a given population, such as patients with COVID-19 or patients with breast cancer. We may also spot changes in the profiles of the patients who had adverse outcomes. Following these initial insights, the subsequent task is to understand *why* such changes happened, the goal of *step 3*.

**Table 1:** Drift types concerning the passing of time, according to Moreno-Torres et al [21]^a^.

Data drift type	Description
Sudden drift	Abrupt and unexpected changes in the data
Incremental drift	Gradual and continuous changes over time
Gradual drift	Slow and steady changes in the data distribution
Reoccurring drift	Periodic or repetitive shifts in the data

#### Semantic Characterization Step

In step 3, the main focus is to learn *why* the changes we observed in step 2 happened. This step integrates fundamental research and business value into our methodology and is heavily dependent on the temporal granularity of the data under evaluation. To the best of our knowledge, this is the first study to examine data drifts in health care from a technology incorporation standpoint. For instance, as mentioned earlier, we may have already learned, as a result of step 1, that a given disease or condition, such as COVID-19, had a decreased lethality over a specific time period. Given this information, what will add value to health care services is the discovery of which repeatable interventions within this time frame can be consistently beneficial.

We begin step 3 by proposing a novel NLP-inspired technique based on token embedding techniques, such as Word2Vec [26], to detect local or individual changes in outcome contexts over time. We opt for NLP-inspired techniques because they effectively model and comprehend “semantics” and “contexts.” In this context, we treat each patient as a “document” and any temporally discrete health care event or information, such as disease codes or items used during a hospital stay, as a “token” (ie, the equivalent of a “word” or a “subword” in NLP). For instance, the underlying premise is that a patient’s semantics can be understood by examining their diseases and consumption history. On the basis of on this representation, we characterize which entities or outcome groups have undergone the most significant changes regarding their defining characteristics in comparison to a baseline or initial time chunk. This assessment assumes a setting where we have an outcome y and the task of predicting this outcome using independent variables X. This characterization can be achieved by comparing the distance of each class’s centroid to a reference centroid, where a “centroid” represents the arithmetic mean of each patient’s features.

The procedure to compute each of these *centroids* is explained in Multimedia Appendix 1. In this figure, we show a simplified view of 2 groups of patients in 2D and how the centroids are calculated to be at the spatial “center” of the groups by averaging their attributes. We can compare different centroids using either a cosine distance or a cosine similarity (equation 1). This type of analysis can guide our research toward a specific hypothesis, filtering down to the pattern changes in specific outcomes, such as death or the need for mechanical ventilation during a hospital stay.



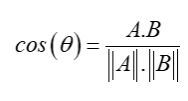



**(1)**

In equation 1, the cosine distance is simply 1 –cosine similarity.

The centroid of each class in the first (time) chunk will be analyzed over time, providing insights into which outcomes (eg, death vs nondeath or hospitalization vs nonhospitalization) underwent the most significant changes. From this observation, we can focus our analysis on the interest group. This approach, which will be further illustrated in our experiments, allows us to compute semantic distances among patients, between patients and outcomes, and among different outcomes.

To apply step 3 to a data set, we need to remember that health care data come in different temporal and semantic granularities. For instance, data sets such as the Brazilian COVID-19 Registry (details presented in the DIS Instantiation for the Brazilian COVID-19 Registry Data Set section) treat each patient as a single data point, characterized by atomic temporal granularity, where temporal effects are observed only at a populational level. In data sets with such low temporal granularity*,* it is as if all events happened simultaneously at the patient level, and we know only the relationship between those events and the patients. In these cases, modeling the relationships between entities and their resulting semantic vectors may require techniques such as graph vectorization.

On the other extreme, data sets with high temporal granularity, such as MIMIC-IV (details presented in the DIS Instantiation for the MIMIC-IV Data Set section), present patients existing within their own timelines, as well as at the populational level. Furthermore, MIMIC-IV has different levels of semantic detail, such as sequential disease codes that could be aggregated into broader groups based on their chapters (eg, both “prostate cancer” and “breast cancer” could be grouped under the “neoplasms” disease code chapter).

In both cases, we would first refer to step 2 to identify suitable candidates for the NLP-inspired modeling. In the case of MIMIC-IV, as demonstrated later, the data show a gradual and trending shift over time, with in-hospital mortality consistently decreasing over the years. Given this pattern and the granularity available in these data, we create sequences of discrete information tokens to elucidate the observed variations for each patient, such as ordinal disease codes or chapters, if a more compact set of possible semantic units is desired.

Finally, we can append “artificial tokens” at the appropriate positions on each patient’s sequence, such as a “death” token at the end of the sequences of deceased patients or an “ICU” token when the patient is transferred to the intensive care unit (ICU), if applicable. With these sequences, we can obtain semantic vectors representing diseases, patients, or outcomes. Following this process on discrete temporal chunks, such as years or months, we obtain distinct outcome tokens for each temporal chunk (eg, “death 2020” and “death 2021,” effectively separating the same outcome over 2 years). With this, it is possible to compare the tokens, examining their relative distance and semantic similarity to each other and other tokens. This allows the identification of what has become more or less similar to the analyzed outcome over time.

Next, we will illustrate the application of our methodology to the 2 aforementioned case studies, with different temporal granularities. The 2 cases are very different in terms of their temporal granularity, volume, and nature of data, demonstrating the generalization capability of DIS.

### DIS Instantiation

We illustrate the application of DIS to analyze temporal shifts by using the MIMIC-IV [18] and the Brazilian COVID-19 Registry data sets [17].

The MIMIC-IV data set is a comprehensive, open-access, and deidentified in-hospital patient record containing sequential diagnosis data; consumption items; vital data records; unstructured eHealth data (text data); and clinical notes for approximately 40,000 ICU patients from 2008 to 2019, designed for research in health care and medical science [18]. In this data set, age is reported in age groups, which is a requirement for deidentification.

The Brazilian COVID-19 Registry is a multicenter retrospective cohort of 10,897 patients with a confirmed diagnosis of COVID-19 admitted between March 2020 and December 2021 from 41 different Brazilian hospitals. For the purpose of the present analysis, variables collected at hospital presentation and at patient discharge were used. The data set consists of >200 features, including known comorbidities, patient’s age and sex, laboratory tests (such as complete blood count, C-reactive protein, and arterial blood gas analysis), vital signs at hospital presentation (ie, arterial blood pressure, respiratory rate, and heart rate), and clinical outcomes [17].

As mentioned earlier, we chose these 2 case studies, as they illustrate scenarios where the available data have very different temporal granularity characteristics, meaning the patient’s timeline can be reconstructed from the data at either a local (individual) or a populational level.

### Ethical Considerations

This study was approved by the Ethics and Research Committee of the Federal University of Minas Gerais (CAAE 70801523.7.1001.5149).

## Results

### Overview

The MIMIC-IV data set comprised 299,712 patients (median age 48, IQR 29-65 years), while the Brazilian COVID-19 Registry data set comprised 10,898 patients (median age 60, IQR 48-71 years).

**Figure 3:**
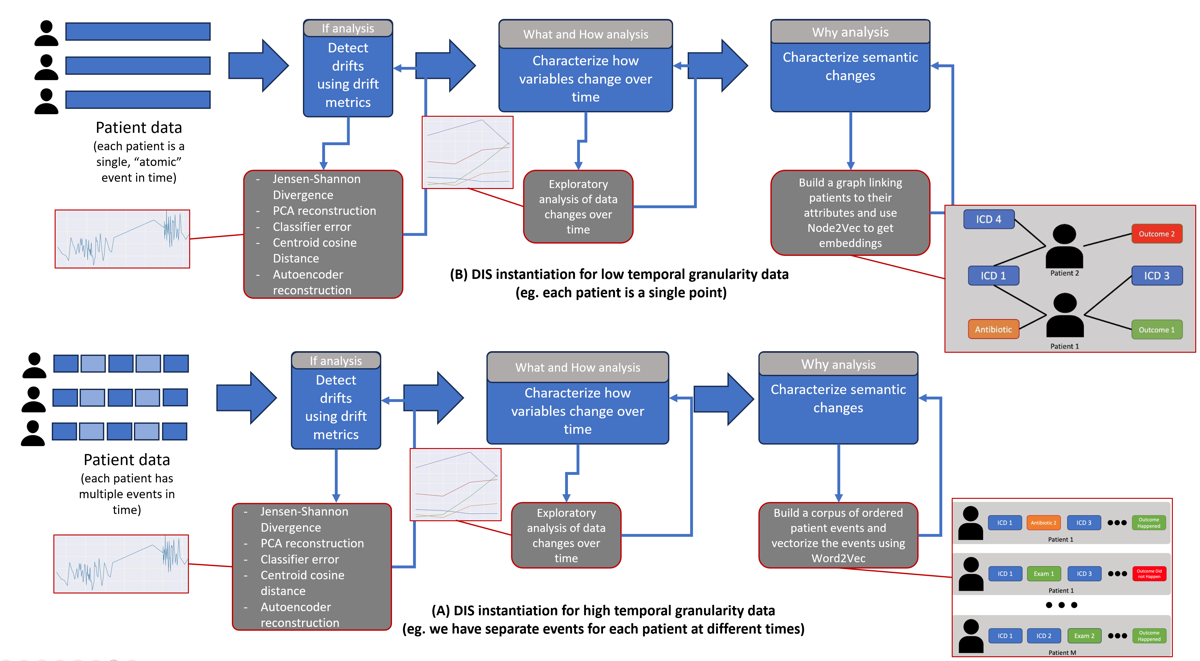
Overview of the instantiation of the detection, initial characterization, and semantic characterization (DIS) methodology to 2 scenarios with different temporal granularities. (A) Medical Information Mart for Intensive Care, version IV (MIMIC-IV) DIS instantiation and (B) Brazilian COVID-19 Registry DIS instantiation. ICD: International Classification of Diseases; PCA: principal component analysis.

Figure 3 illustrates how the DIS methodology is instantiated concerning the data’s temporal granularity for each scenario. As explained, DIS consists of 3 steps (detection, initial characterization, and semantic characterization). The temporal granularity of the available data affects specifically the last step (semantic characterization). The figure also shows that several methods can be applied for the detection step. In our experiments, we tested and compared 5 different methods regarding their capability of accurately identifying temporal drifts in the detection step. In the second step, different exploratory techniques that measure the relationship between the dependent *P*(*y*) and independent *P*(*x*) variables over time can be used. We exploited multiple alternative techniques, such as feature importance and Pearson correlation. Finally, in the last step, our aim was to generate semantic embeddings for outcomes and other health care events over time and to derive insights from comparing these embeddings. We tested two different alternatives for producing such insights: (1) using our semantic embedding modeling and (2) using traditional clustering techniques over the untreated (original) data without the semantic treatment. The goal of using these 2 techniques was to illustrate insights that can be obtained with the semantic layer, which would be difficult to obtain otherwise.

### DIS Instantiation for the MIMIC-IV Data Set

A notable characteristic of this data set is its high temporal granularity, enabling the tracking of time progression within each individual’s hospital stay. High temporal granularity means we know the sequence of health care events at the individual level. This facilitates obtaining invaluable insights into the relationships between such events, much like it helps us learn about the semantics of words in NLP. It has been consistently shown that the order of precedence between words and how often they appear with other words are representative of those words’ semantics [26]. We claim that the order of precedence and cooccurrence between health care events can also contain the “semantics” of those events. A distributed representation built from these relationships could cluster similar health care events, such as the representation of different types of diabetes or hypertension and their associated complications, in close proximity in the space. Although all dates in the data set are anonymized for privacy reasons, we can track each individual’s sequence of events using the provided masking of dates. This date masking is consistent in a manner that allows for time tracking during each patient’s hospital stay, and it contains a special attribute that allows for the association of patients with the yearly interval during which they were hospitalized. These yearly interval data allow us to compare how patients in each year group behaved as a group, meaning we can also measure temporal effects at the populational level. The period covered by these data set ranges from 2008 to 2019.

In other words, the data set offers temporal granularity at both the population and individual levels. However, breaking this data set into arbitrary temporal chunks is challenging because the dates are masked. Despite this, the data set contains a nonmasked anchor year group that assigns each patient to an actual year interval during which they were hospitalized. Multimedia Appendix 2 explains how this variable works. Essentially, a random time delta is fixed for each patient and added to all relevant dates, effectively masking them while preserving the relative time intervals for that patient. Consequently, direct comparison of dates between 2 different patients is not feasible, except for their “anchor_year_group” variables. For instance, a patient hospitalized in 2015 may have (through the added random time delta) dates that appear later than those of a patient hospitalized in 2020. We can only directly compare dates within the context of each patient. The real year interval during which each patient was hospitalized is preserved in their ”anchor_year_group“ variable, which we use in all chunking for this data set henceforth.

### DIS: Detection Step (MIMIC-IV)

As described, the temporal chunks in MIMIC-IV were given by the “anchor_year_group” variable. We used this variable to separate patients into the 4 groups provided within the data set. We then used alternative drift detection approaches, namely Jensen-Shannon divergence, autoencoder reconstruction error, PCA reconstruction error, centroid distances, and classifier prediction error in separating time chunks plot for this data set considering in-hospital ICD diagnosis. The Jensen-Shannon divergence formula is shown in equation 2, where *KL* is the Kullback-Leibler (KL) divergence [27], and *P* and *Q* are the 2 variables being compared.

**Figure 4:**
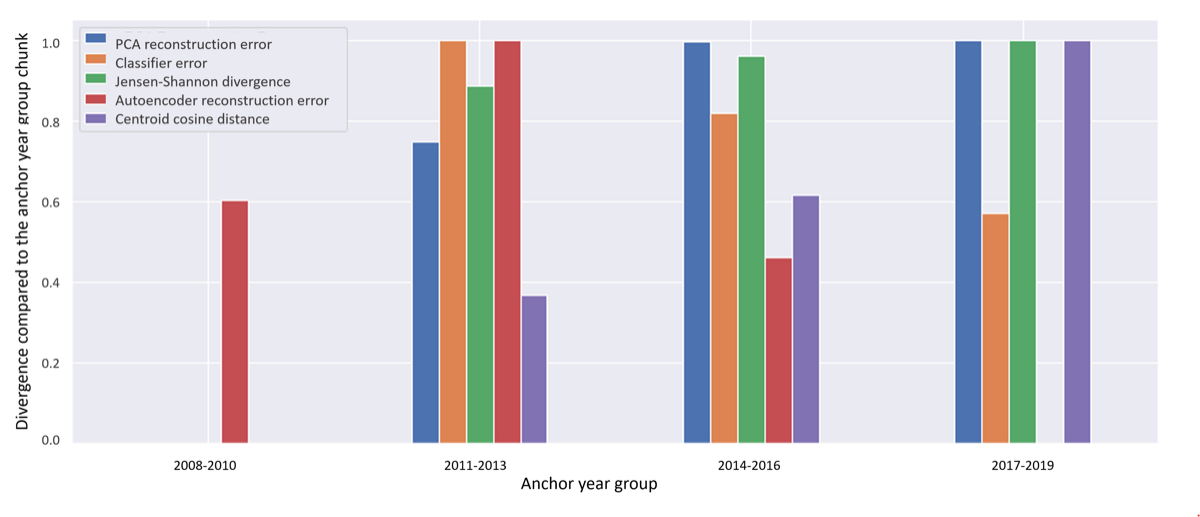
Different drift detection metrics over time on the Medical Information Mart for Intensive Care, version IV (MIMIC-IV) data set, considering in-hospital International Classification of Diseases (ICD) diagnosis. PCA: principal component analysis.

We started step 1 of DIS with the *drift detection* substep. As previously described, the temporal chunks in MIMIC-IV were identified through the “anchor_year_group” variable. We used this variable to separate patients into the 4 groups provided within the data set. Figure 4 shows the Jensen-Shannon divergence plot for this data set considering in-hospital *ICD* diagnosis. The Jensen-Shannon divergence is shown in equation (2), in which *KL* is the KL divergence, *P* and *Q* are the 2 variable distributions being compared, and we compute na average of each possible KL divergence combination between the two distributions. Since the KL divergence is asymmetric, the calculation described can be interpreted as a symmetric divergence between the two distributions. This metric was tracked to evaluate whether the data distributions changed over time, how fast they changed, and whether the data shift was temporary.

*JSD*(*P||Q*) = 1/2 *KL*(*P||M*) + 1/2 KL(*Q||M*) **(2)**

In equation 2, *KL* is the KL divergence, *M* is 1/(*P *+ *Q*), and *P* and *Q* are the distributions of the variables we compared.

Figure 4 presents the results of our drift detection metrics, applied to the various “anchor_year_groups” in the MIMIC-IV data set. The figure depicts the normalized magnitude of the drift signal calculated per “anchor_year_group.” The drift signals were normalized in the 0 range for visualization, as shown in equation 2. The results for the Jensen-Shannon divergence, PCA reconstruction error, and centroid cosine distances revealed a trend toward increasing distance between the variable distributions over time, which did not revert to prior levels, suggesting a gradual temporal shift. As seen in Multimedia Appendix 2, this drift occurred gradually over several years, with a more pronounced change between the first 2 temporal chunks.

By contrast, when examining the autoencoder reconstruction error and classifier error metrics, a peak divergence was observed in the second time chunk (2011-2013), which gradually trended toward the baseline. As models with more parameters, these 2 drift metrics were sensitive to a combination of the data distribution, novel data points (ie, rare diseases or diseases not present in the reference time slice), and numerical outliers in the case of the autoencoder reconstruction error. For example, the disease codes appearing in the second chunk had the smallest intersection with the reference chunk, meaning they had the fewest diseases occurring concurrently in both chunks. This likely explains why the autoencoder reconstruction error and classifier error metrics exhibited their highest peaks in this slice.

In summary, the Jensen-Shannon divergence metric yielded more robust drift signals in our tests. It is important to note that the best metric depends on the most relevant type of drift for the data collection being analyzed. The Jensen-Shannon divergence is robust at detecting distribution changes, just as robust as the classifier error metric. If we are interested in detecting the occurrence of outliers or novel samples not seen before, the reconstruction errors might result in better detection. The choice of metric must be informed by the characteristics of the metrics themselves as well as the characteristics of the data stream being monitored.

NormalizedSignal = (X – min[X])/(max[X] – min[X]) **(3)**

As show in equation 3, normalization is used to calculate the normalized magnitude of the drift signal.

### DIS: Initial Characterization Step (MIMIC-IV)

After establishing that a drift has indeed occurred, especially based on the results of the most accurate method, the Jensen-Shannon divergence, we proceeded to *step 2.* In this step, we strived to understand how the independent variables (*P*(*X*)) affect the outcome, which is our *dependent variable (P(y|X)),* and how the relationship between dependent and independent variables changes over time. This analysis can be accomplished by examining changes in correlations and feature importance over time, as well as by characterizing the distribution of different features over time. For instance, in Multimedia Appendix 3, we show how the relative distribution of the “death” outcome has changed over time in this data set. This means that our data exhibit a consistent trend toward in-hospital mortality reduction over time, which indicates a change in the relative distribution of the 2 possible categories (deceased × not deceased) for this outcome.

**Figure 5:**
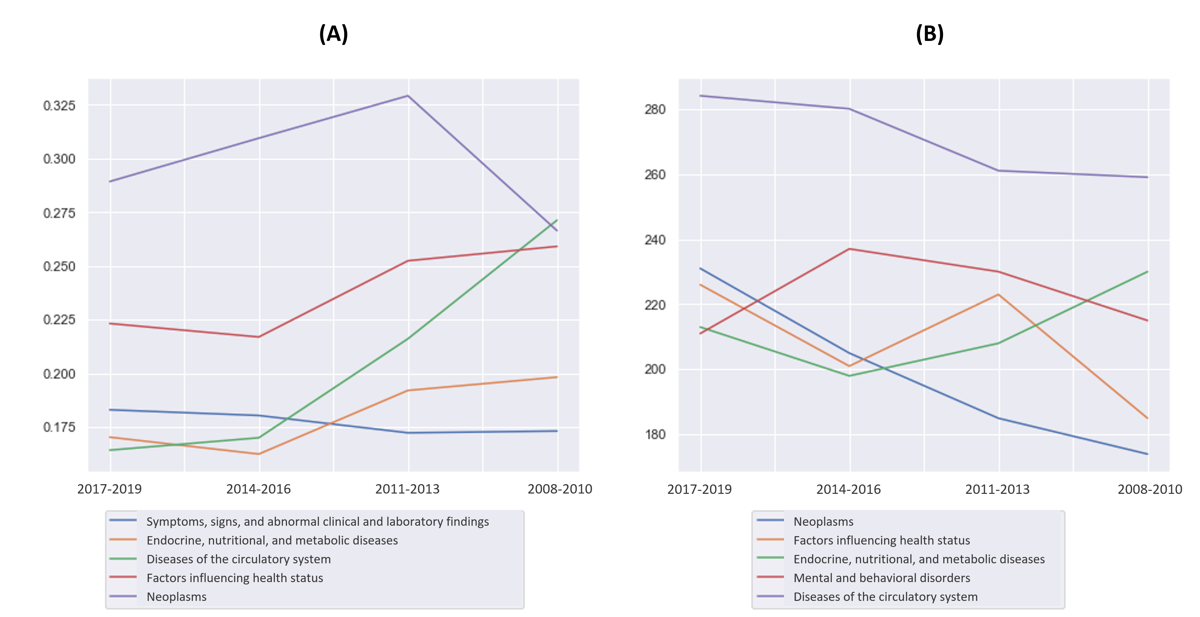
(A) Pearson correlations between the top 5 International Classification of Diseases (ICD) chapters (according to ICD-10) most correlated with the death outcome over time. (B) Feature importance among the top 5 ICD chapters most predictive of the death outcome over time.

In Figure 5, we show the correlations and feature importance variations of the top 5 most correlated and the top 5 most predictive *ICD* chapters (according to *ICD-10*) and the “death” outcome (according to feature importance). For instance, Figure 5A shows some expressive variations, such as how circulatory system diseases seem to grow more correlated with death over time, Figure 5B shows how neoplasms seem to become less predictive of death over time.

In Multimedia Appendix 4, we show how the different outcome groups behave over time from given baseline, in particular the patterns of independent variables given the outcome categories P(y|X) observed in the first temporal chunk. To obtain this result, we computed the arithmetic mean of each class’s features in each “anchor_year_group” and calculated the cosine distance between these means over time, taking the first chunk as a reference to compare all other chunks against it. In this particular figure, we represent each patient as a “corpus” containing all their health care events (such as diseases and medications used during the hospital stay), then encode each feature as a 1-hot sparse matrix (each event can have the value “0,” if it did not happen for a particular patient, or “1,” if it did), and subsequently average these features. Notably, this representation treats each patient as a “bag of health care events,” disregarding the order of precedence between those events, unlike what we did in our semantic characterization step. In the specific case, we show how the “death” outcome exhibits greater temporal drifts over the available time chunks in both data sets compared to the overall hospitalized patient population.

### DIS: Semantic Characterization (MIMIC-IV)

**Figure 6:**
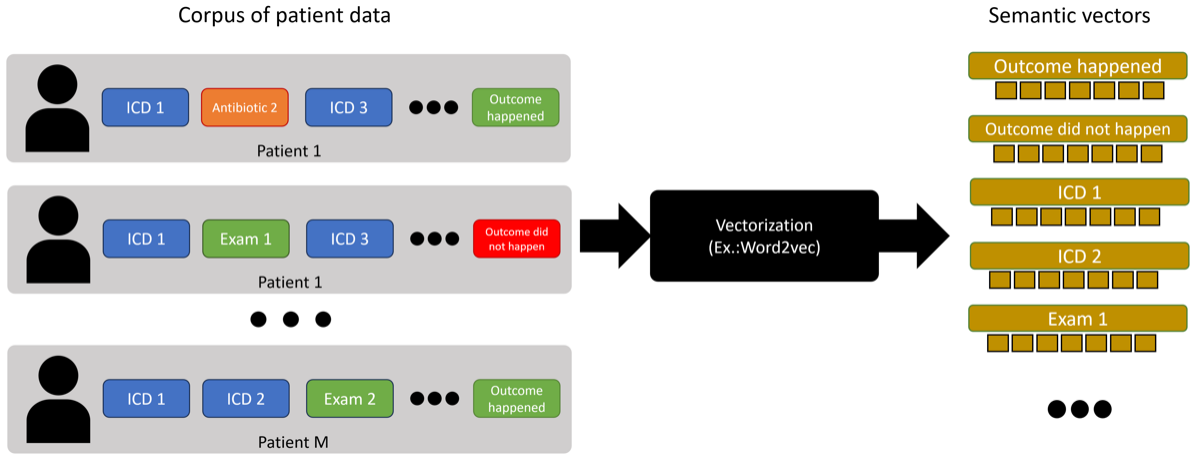
How to generate semantic vectors? We start by generating a corpus of temporally ordered patient discrete data points. Then, we vectorize the tokens of this corpus using Word2Vec to obtain semantic vectors for dependent and independent variables. ICD: International Classification of Diseases.

In Table 2, we show the top 5 *ICD-10* chapters that have become more and less similar to the “death” outcome over time. Notably, certain diseases, such as neoplasms, have become less similar, while others, such as malformations and circulatory system diseases, have become more similar. That is consistent with the findings in step 2, and over the next few paragraphs, we describe the procedure to obtain this similarity score. We explain the token-level vectorization process for both dependent and independent variables in Figure 6. First, we compiled a temporally ordered list of patient data, consisting of discrete data points such as items consumed during hospital stay (antibiotics, anti-inflammatories, etc), disease codes (using *ICD*), and procedures. At the end of each patient’s sequence, we appended the outcome category for that patient. To classify the outcome, we divided binary outcomes into distinct tokens, such as “deceased” and “not deceased,” and used the corresponding token to generate our training corpus. Continuous outcomes and dependent variables could be binarized using a simple histogram binarization scheme, as demonstrated in the next analysis. Following the corpus generation, we used it to train token embeddings with Word2Vec [26]. This method produced embedding vectors for both dependent and independent variables, allowing semantic comparisons between different entities, such as the ”death“ outcome and different disease codes. We created 1 outcome token for each outcome category and temporal chunk in our data set. This allowed us to evaluate how an outcome such as “death” may have drifted closer to or farther from certain diseases or procedures over time.

In Multimedia Appendix 5, we show the top 5 conditions that became more similar to the “death” token and the top 5 conditions that became less similar when comparing the first and last time chunks. Since every entity is a “token,” we could evaluate similarities between diseases and disease chapters, between patients and diseases they have not yet been diagnosed with, and between outcomes and diseases (Multimedia Appendix 5). In particular, in Multimedia Appendix 6, we demonstrate changes in similarity for the “dysphagia following stroke” *ICD* code within the MIMIC-IV data set [18]. Our analysis revealed a rise in the simultaneous appearance of ICD codes related to obesity between the periods of 2011 to 2013 and 2017 to 2019. This trend aligns with broader observations indicating an uptick in obesity rates across the United States. Importantly, it is essential to recognize that this method does not permit the establishment of causal relationships; rather, it emphasizes changes in correlation and cooccurrence.

The *step 3* analysis can also be conducted at different levels of granularity to gain a deeper understanding of the observed changes. From step *2*, it can be inferred that mortality has been decreasing and has some relationship with particular disease groups. If *step 3* is performed at the disease code level, as shown in Multimedia Appendix 6, chapters that had considerable shifts in their similarity to the “death” outcome, either increasing or decreasing similarity, can be identified. For instance, the findings confirm what is illustrated in Figure 6, where “cancer” shows a decreasing similarity to the outcome, while the variable “circulatory diseases” exhibits an increasing similarity to the outcome. This observation is further supported by the results shown in Multimedia Appendix 7, where an absolute increase in the number of patients with cancer over time is shown, associated with a relative decrease in in-hospital cancer-related deaths between 2008 and 2019.

To further illustrate how the proposed DIS semantic analysis based on embedding distances among entities of interest can help in better comprehending the reasons for the drifts, we contrasted the previous analyses of our third step with a traditional clustering analysis for the MIMIC-IV data set. This analysis used a syntactically oriented term frequency–inverse document frequency (TF-IDF) [28] representation for the entities, built from the same corpus of clinical entities. In a TF-IDF representation, each dimension corresponds to a unique term (word) in the document corpus. The value in each dimension reflects the importance of that term in a specific document, calculated by multiplying the term’s frequency in the document (term frequency) by the inverse frequency of the term across all documents (inverse document frequency). In our case, each “document” was a patient, and each “word” was a health care event, such as the identification of a novel disease. We applied a spectral clustering [29] procedure to the TF-IDF representation of the entities to create the clusters. The results are shown in Figure S8. To obtain the 4 clusters displayed in Multimedia Appendix 8, we used a silhouette analysis using 2 to 15 clusters.

Multimedia Appendix 8 shows the top 5 most frequent diseases for each of the 4 clusters (y-axis). On the x-axis, we present the index of each cluster. Multimedia Appendix 8 shows how the relative frequency of each cluster changed over each “anchor_year_group.” A few points stood out from the clustering analysis illustrated in Multimedia Appendix 8. As it can be observed, the cluster analysis using syntactically oriented vectors made it harder to interpret the drivers of a data drift when compared to DIS. For instance, some semantically similar diseases, such as “other and unspecified hyperlipidemia” and “hyperlipidemia, unspecified,” may have very distinct profiles in different clusters, such as in clusters 0 and 2, each having a high concentration of patients with either one of these diseases. The main problem of this particular cluster analysis based on syntactically oriented representation is the separation of semantically similar entities into distinct clusters. In DIS, similar entities will be represented similarly and thus analyzed in conjunction.

**Table 2:** Change in similarity by ICD^a^ chapter.

*ICD* chapter	Change in similarity	Direction
Diseases of the nervous system	–0.14	Less similar
Diseases of the musculoskeletal system	–0.12	Less similar
External causes of morbidity and mortality	–0.10	Less similar
Diseases of the digestive system	–0.08	Less similar
Neoplasms	–0.02	Less similar
Congenital malformations	+0.40	More similar
Diseases of the circulatory system	+0.35	More similar
Diseases of the genitourinary system	+0.30	More similar
Endocrine, nutritional, and metabolic diseases	+0.25	More similar
Diseases of the skin and subcutaneous tissue	+0.20	More similar

^a^ICD: International Classification of Diseases.

### DIS Instantiation for the Brazilian COVID-19 Registry Data Set

The median age was 60 (IQR 48-71) years, and 21.72% (2367/10,898) were women (5012 patients). In this data set, 21% of registered patients died, yielding an unbalanced classification problem when predicting future deaths. The data set has low temporal granularity, with only 1 data point per patient, which precludes time tracking during hospital stays. Consequently, we could measure time only at the populational level. In other words, unlike the previous case study, there was a single “snapshot” for each patient, with no temporal evolution at the individual level.

### DIS: Detection Step (Brazilian COVID-19 Registry Data Set)

As in the previous case study scenario, we evaluated the same 5 alternative techniques, namely the PCA reconstruction error, autoencoder reconstruction error, classifier error (in separating past vs future), and Jensen-Shannon divergence. All these metrics measure the drift compared to a reference temporal slice and do not require setting a specific outcome or using labeled data.

**Figure 7:**
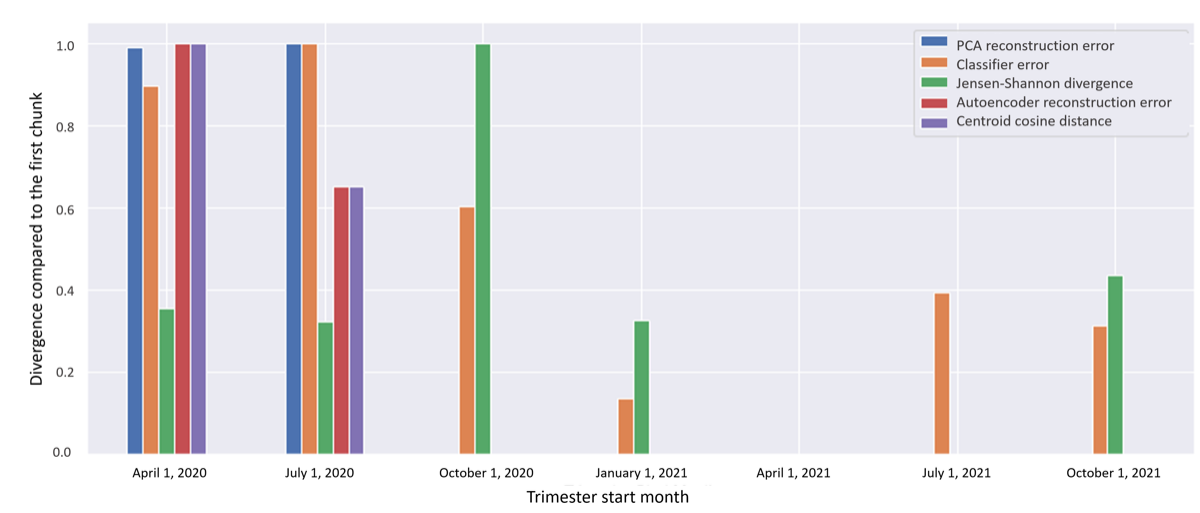
Different drift detection metrics over time in the Brazilian COVID-19 Registry data set. PCA: principal component analysis.

The outcomes of this procedure are illustrated in Figure 7, where the divergence sharply increases starting from the final quarter of 2020, based on the Jensen-Shannon divergence metric. Numerically, a drift is indicated in this interval as the divergence levels surpass a user-defined threshold, such as a fixed threshold of 2 SDs or a threshold informed by domain expertise. As depicted in the figure, the PCA reconstruction error, autoencoder reconstruction error, and centroid cosine distances indicate positive drift signals in the quarter starting from April 2020. During this semester, the Brazilian COVID-19 Registry data set exhibited a small number of numeric outliers, which were identified by these methods. Conversely, the Jensen-Shannon method signaled a data drift in the quarter starting from October 2020, which aligns with the “official” start of the second wave in Brazil in November 2020. Meanwhile, the classifier error method indicated a drift in July 2020, which falls between the identification of numeric outliers and the actual distribution change from the first wave to the second wave. Both the Jensen-Shannon method and the classifier error method signaled drift closer to known actual changes, while the other, more reconstruction-based methods were more sensitive to numeric shifts, which were not necessarily associated with changes in the underlying distributions.

### DIS: Initial Characterization Step (COVID-19)

Once a drift was detected, we proceeded with the second DIS step, initial characterization. This step aims to understand the main drivers (“what”) of changes during the considered period and “how” they affect the underlying outcomes. At a high level, this begins with the characterization of the changes in the outcome (the independent variable) over time. In Figure 7, we illustrate this upon evaluating the variation in COVID-19–related mortality in our data set. This example displays a trend toward a reduction in the death outcome over time. At the initial characterization step, it is expedient to examine the distribution of the outcome of interest (death, ICU admissions, etc) as well as those of the most predictive independent variables (eg, those with the highest correlation with the desired outcomes or higher feature importance in a tree-based classifier).

To guide the next steps, it is helpful to check how much each outcome category’s properties (such as the mean age of the deceased patient population or the prevalence of hypertension) have changed over time. In particular, focusing on which outcomes have changed the most helps target specific subsets of the data that could better explain the observed phenomena. We show an example in Multimedia Appendix 9, where we analyzed such variations in the Brazilian COVID-19 Registry data set. To build the graphs in this figure, we split our data set into time chunks. For each chunk, we separated all patients into classes according to their outcome (eg, dividing the population into deceased and nondeceased and then representing the chunk by averaging all of the patient’s features in each category). For each subgroup of patients within the same time chunk and sharing the same outcome, we computed the centroid of that class (the arithmetic mean of all attributes). We then took the first chunk as a reference and compared each class’s chunk arithmetic mean to the reference using a cosine distance. Multimedia Appendix 9 shows how much the deceased patients’ characteristics changed more than those of the overall population during the same period.

**Figure 8:**
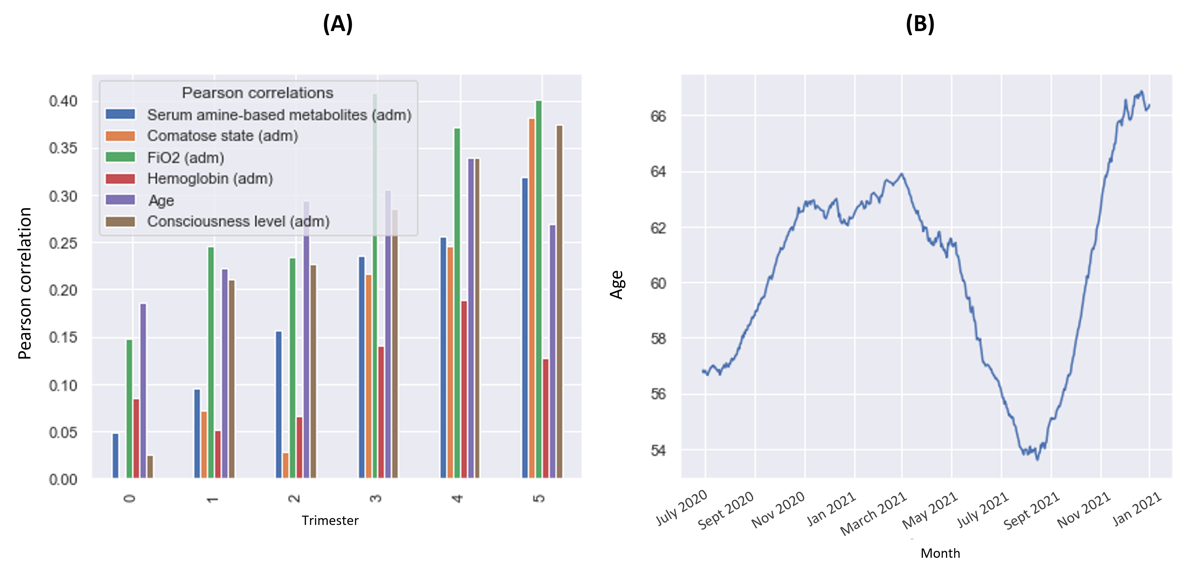
(A) Pearson correlations over time for the overall top 6 most predictive variables in the Brazilian COVID-19 Registry data set. (B) Median age of hospitalized patients dying from COVID-19.

A better comprehension of the drift drivers during the COVID-19 pandemic emerges from Figure 8. As shown in Figure 8A, we observed how the overall best predictors of death changed over time through Pearson correlation analysis conducted each trimester on the data set. At the beginning of the pandemic, age was the single best predictor of death, in trimesters 0 and 2. As the vaccination campaign started, older adults were prioritized and received immunization first. This led to a progressive deterioration of the predictive value of age, as well as an overall decrease in mortality (Multimedia Appendix 10), culminating in the latest trimester, where age was the worst predictor among the top 5 variables. In Figure 8B, it can be seen that the median age of the deceased patient population over time.

In summary, the second step revealed that the COVID-19 data showed a progressive decrease in patient mortality (Multimedia Appendix 10), with a more pronounced change in the group of deceased patients (Multimedia Appendix 9). It was also possible to notice that the overall characteristics of the patients who were dying changed abruptly (Figure 8). From the remaining characterization steps in Figure 8, we can see that age lost its predictive capacity (Figure 8A) over time, while clinical features such as the patients’ fraction of inspired oxygen (FiO_2_) became better predictors of death. Concurrently, there was a reduction in the median age of patients who were dying (Figure 8B).

### DIS: Semantic Characterization (COVID-19)

Following the conclusions from the previous step, we moved further into the semantic characterization step. As the Brazilian COVID-19 Registry data have low temporal granularity and most of their features are continuous, what requires data categorization to enable the use of NLP techniques to treat words and other semantic units.

Subsequently, due to low granularity at the individual level, we needed to model relationships between these now-discrete entities. In more detail, we assumed that the temporal precedence between events imposes a relationship between them and that this relationship can be learned and embedded into a distributional representation. The issue with low temporal granularity data is that the order of precedence is not known; hence, it is not possible to model it directly. Therefore, we modeled all health events (from the perspective of a single individual) as if they happened simultaneously. Therefore, in this setting, we modeled the passing of time only from the perspective of the population and not from the perspective of the individual. This means that we only knew, for instance, that a given patient had events (such as new diseases or use of medications) 1, 2, and 3, but we did not know the order of precedence between these attributes, something that was explicit in the MIMIC-IV data due to high temporal granularity. We began by discretizing the continuous features with a histogram discretizer, which essentially breaks the data intervals into “equal width segments” and then assigns a “token” (ie, a string or integer value) that is unique to patients having that attribute in that specific range of values.

**Figure 9:**
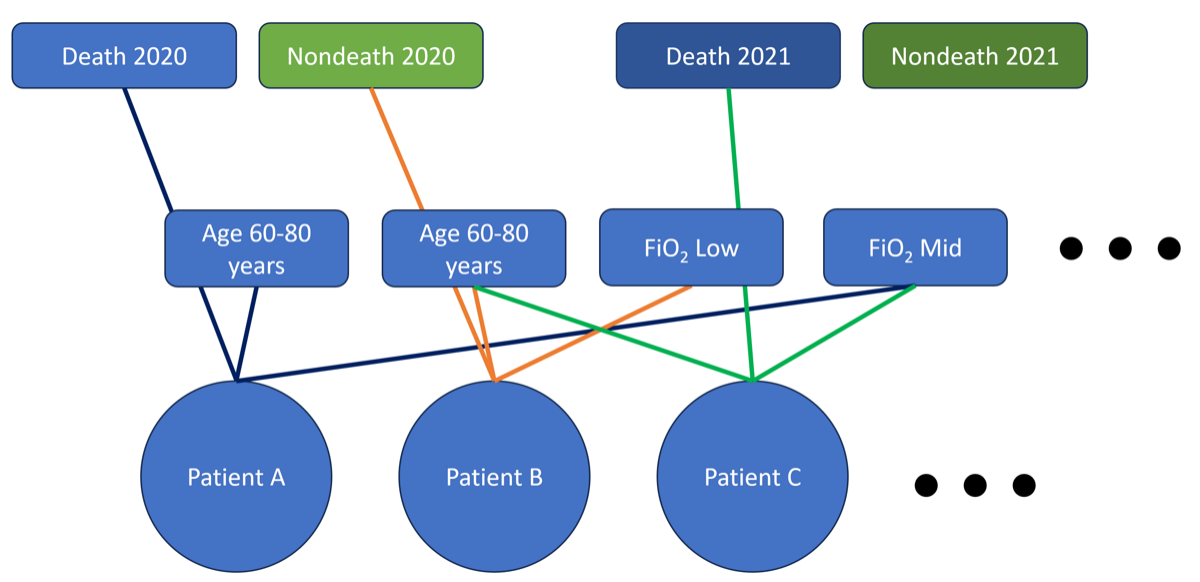
Example of how to create a patient graph with tokenized dependent variables and temporal outcome tokens.

**Figure 10:**
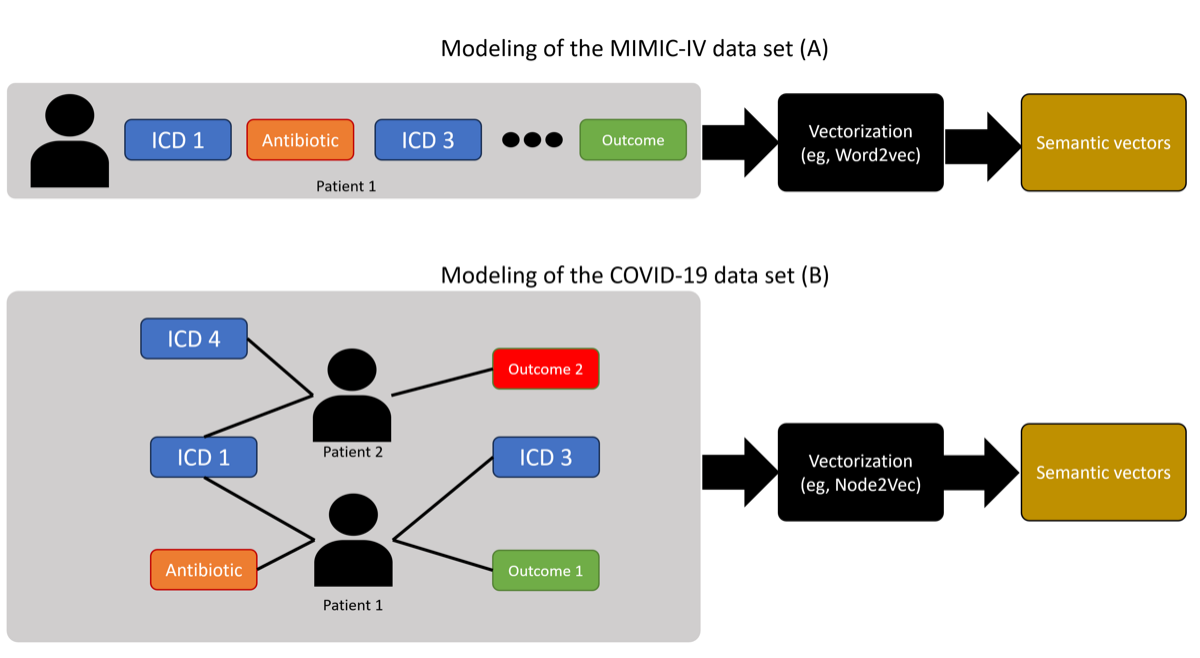
(A) Modeling of the Medical Information Mart for Intensive Care, version IV (MIMIC-IV) data set as an ordered sequence of patient tokens. (B) Modeling of the Brazilian COVID-19 Registry data set as a graph connecting multiple patients through their common token. ICD: International Classification of Diseases.

After that, we created a graph with patients, discretized continuous attributes, discrete attributes, and outcomes, such as the one in Figure 9. To build this graph, we connected each patient to their attribute tokens and outcomes while creating one outcome token for each time chunk under analysis. Finally, we embedded the graph using a node embedding algorithm such as Node2Vec [30]. We contrasted this procedure with the one adopted to characterize the MIMIC-IV data set (Figure 10). As discussed before, in MIMIC-IV, the temporal order is defined at the individual level, with entity relationships determined by the timeline. By contrast, the Brazilian COVID-19 Registry data set presents events as occurring “simultaneously” at the patient level, limiting the understanding of relationships between events and patients. In this case, to derive semantic vectors representing entity relationships, we approached it as a graph vectorization problem.

To analyze the resulting model, we compared the outcome embedding vectors to evaluate their similarity to each other and to other patient attributes. We show the results of this procedure in Multimedia Appendix 11. From that, it is evident that the 2021 death outcome token increased in similarity to lower age groups, such as age groups 18 to 39 years and 40 to 61 years, while decreasing in similarity to older age groups, such as age groups 62 to 83 years and 84 to 105 years. This observation further validates the previous findings and introduces new elements not captured in earlier steps. We could also see an increase in similarity to lower admission heart rates and lower admission serum sodium values, as well as lower FiO_2_ at admission, showing a shift in disease severity markers over this time frame.

As mentioned earlier, for comparative purposes, to emphasize the semantic capabilities of the proposed DIS procedures, we compared our semantic-step results with those obtained through traditional clustering analysis for the Brazilian COVID-19 Registry data set (Multimedia Appendix 12). In this analysis, entities were represented by a syntactically oriented TF-IDF representation. In Multimedia Appendix 12, we show the top 5 highest-value features for each of the 6 clusters selected using the silhouette analysis, as was done for the MIMIC-IV case. In Multimedia Appendix 12, we show how the relative frequency of each cluster changed over time in each trimester.

Similar to the MIMIC-IV case, the clustering analysis of the COVID-19 data was not as straightforward to interpret as the DIS analysis when searching for the drivers of data drift. For example, we identified a cluster of patients who underwent a “transplant” and another cluster of patients with diabetes mellitus type 2, but the reasons why these particular clusters were selected and the reasons for the drifts could not be easily derived through a straightforward analysis of these syntactically oriented clusters.

## Discussion

### Comparison With Prior Work

Multiple studies have analyzed variations observed over time in class distributions and model effectiveness and their overall impacts. Studies such as Salles et al [31] and Mouro et al [32], for instance, performed a detailed characterization of such effects in textual data sets of documents organized into topics. Health care data, however, are quite different from simple text data [31,32]. To begin with, this type of data is multimodal, including tabular and sequential information in the form of vital measurements, disease code diagnosis, and items consumed during a hospital stay, as well as common text, images, wave forms, and sometimes even sound waves. Furthermore, the data may experience sudden and specific drifts driven by new medications, vaccines, surgeries, and public policies [9]. For example, an effective vaccine may cause the eradication of a disease, resulting in a subsequent data drift [33]. While most studies on health care data focus on either drift detection or drift adaptation [33,34], our work is unique in that it focused on drift detection, monitoring, and characterization. We advanced the existing literature by leveraging these 3 steps to pursue explanations for health care data drifts.

Concerning terminologies and problem-setting definitions, Gama et al [35] defined data changes as being related to the distribution of the independent variables P(X) and dependent variables P(y) or the conditional probability of dependent variables for given independent variables P(y|X). Works unified and consolidated some of the underlying terminologies [21,36]. As defined by Lu et al [36], data and concept drifts can be categorized based on how they behave over time, being (1) sudden (ie, 1 event permanently changes the “meaning” of a concept), (2) incremental (ie, 1 event incrementally generates gradual changes to the ”meaning” of a concept), (3) gradual (ie, the concepts interchange gradually until the complete shift occurs), or (4) reoccurring (ie, a transient concept drift).

Approaches to detect and learn in the presence of concept drifts do exist. However, in most contexts, naively monitoring data drifts may be expensive, as it often requires data labeling. As an alternative approach, Haque et al [37] used an ensemble of classifiers to report their prediction confidences and monitor changes in their confidence distribution to detect when a concept drift occurred. In the data sets used in this paper, however, deaths are readily available labeled data, which means that our main issue was related to learning in the presence of a data drift.

A common approach to drift detection is monitoring model outputs, as in the study by Sahiner et al [38]. These “model monitoring” approaches are not always possible or desirable; for instance, Tiwari and Agarwal [39] argued that labels are a resource that is not always available and suggested exploring other options, such as detecting drifts by monitoring changes in the underlying data distributions. Following this idea, we propose a *drift monitoring* procedure that is independent of labels and focuses on distribution changes over time. Additionally, Tiwari and Agarwal [39] provide a comprehensive review of useful health care data type classification and data drift management strategies in data streaming scenarios. Textbox 2 details the categorization of health care data proposed by these authors.

In addition to the categorization mentioned in Textbox 2, Tiwari and Agarwal [39] discussed the use of sampling in diverse forms to handle data streams and drifts. In health care data, it is common to encounter massive data sets encompassing multiple years and thousands of patients. For such cases, sampling may be a viable option. Given the size and nature of our data sources, we opted to work with the complete data set available instead of using sampling. The decision to use sampling should be evaluated depending on the type of ML algorithm used, the available computing capabilities, and the data set size.

Drift detection has multiple beneficial impacts on health care. Once detected and treated, a drift can be used to help maintain and enhance model effectiveness. Additionally, it can be useful to detect whether a new treatment is changing the outcomes of a disease in a meaningful manner or even understand populational trends to derive health policies. A recent example is the COVID-19 pandemic. This topic was explored in the studies by Jung et al [41] and Jassat et al [42], which showed differences in hospitalized patient profiles as new COVID-19 waves spread. Another study has explored how the death prediction task evolved throughout the pandemic, showing that factors such as vaccination changed the profile of patients who were severely ill [4]. These characterizations can help in the detection of important pandemic events, such as the impacts of vaccination, the emergence of new COVID-19 strains, and the emergence of new viral strains resistant to current therapies. In this context, we focused our characterization efforts on technology evaluation through the lens of data drifts in a health care setting.

Some solutions have been reported in the literature to address the challenge of learning in the presence of data drifts, and most of these solutions focused on sample selection or sample weighting, with variations on how they derive the final weighting or sampling. Klinkenberg [43], for instance, tackled the problem by using support vector machines for both sample selection and sample weighting, using an iterative process that sequentially trains support vector machines to find the training instances that constitute the model’s support vectors [43]. Kolter and Maloof [44] used a special weighted ensemble to learn in the presence of such drifts. Salles et al [6,31,45] used a temporal weighting function that can be automatically learned to select relevant samples for each training window. Finally, Rocha et al [7] tackled the problem using temporal contexts. The authors analyzed document collections that evolved over time and defined a temporal context as portions of documents that minimize the temporal effects of class distribution, term distribution, and class similarity over time. This method is used to devise a greedy strategy to optimize the trade-off between undersampling and temporal effects. We were inspired by this latter work in our methodology. Most of these approaches, however, are not applied to the health care setting, focusing mostly on common text data.

Another relevant setting is detecting drifts in data streams. This is potentially relevant to some health care data, especially sensor data, which are most commonly obtained from hospitalized patients but also streamed from personal health devices such as smartwatches and heart rate sensors. Zliobaite et al [46], for instance, proposed a continuous loop of labeling new samples under a labeling budget and used active learning to detect data drifts.

Class imbalance is another important aspect of detecting data drifts in health care data. Disease occurrence is naturally unbalanced, with common diseases such as diabetes or hypertension affecting between 5% and 30% of the population [47,48]. Rare diseases, by contrast, have a prevalence in the order of <10 patients per 100,000 or 1,000,000 inhabitants, with combined prevalence among all rare diseases being estimated to be between 3.5% and 5% [49]. Most approaches to handling such class imbalances in the data drift literature focus on oversampling, undersampling, or a combination of both. Gao et al [50], for instance, proposed oversampling the minority class over multiple time slices while undersampling the majority class using only the most recent slice. Ditzler and Polikar [51], by contrast, focused on using incremental learning combined with the synthetic minority oversampling technique [52] to learn a classification ensemble that can deal with both the class imbalance and concept drifts in streamed data. In particular, the combination of models and data sets used in our work was robust to such class imbalance issues and did not require using these types of techniques, as discussed in the following sections.

**Categorization of health care data.**

**Categories**

1. Clinical data, such as the records in Medical Information Mart for Intensive Care, version IV (MIMIC-IV) [18] and the Brazilian COVID-19 Registry [17], are desirable if the goal is to describe data drifts related to the impact of specific interventions, such as the introduction of a new drug or therapy.
2. Self-administered data, obtained from questionnaires, usually investigate lifestyle variables, such as smoking or alcohol consumption habits.
3. Biological data, usually obtained by measuring parameters in biological samples such as blood and urine, are often the result of a laboratory study.
4. Molecular data are the kind of data encoded in protein databases such as UniProt [40], genome databases, or even drug-to-molecule interaction databases.
5. Exposure data encode patients’ exposure to given events, drugs, or interventions.
6. Modeling data are data generated from models, including estimated risks given the patient’s exposure.

### Summary of the Main Results of Applying DIS to the MIMIC-IV Data Set

The instantiation of the drift detection step using several distribution comparison metrics showed the flexibility of the methodology. It also demonstrated that, for the purpose of separating the temporal chunks in this particular scenario, metrics such as the Jensen-Shannon divergence or the classifier errors capture the underlying distributions better than particular outliers or novel samples. Higher values in these metrics imply more significant “populational” changes, such as a gradual shift in the composition of the in-hospital population’s disease burden.

As seen in the drift detection step (Table 2), there is a gradual but persistent pattern in MIMIC-IV, happening over several years. This gradual change may be caused by various factors, such as an increased tendency for patients who are terminally ill to receive end-of-life care at home or advancements in therapeutic techniques for certain diseases. The nature of the expected data change can be hypothesized based on characteristics such as the suddenness or gradualness of the drift, its persistence, and its duration, along with the results from the next analytical steps in DIS. This difference becomes evident when comparing the MIMIC-IV and the Brazilian COVID-19 Registry data sets.

The initial characterization step (Figure 5) revealed a trend toward a decrease in overall mortality over time, and this is the “context” in which we interpreted subsequent findings. Additionally, Figure 6 indicates that the overall characteristics of the deceased patients changed more than those of the overall in-hospital population over the observed time frame. This means that the reduction in overall mortality is due to changes in the characteristics of the patients who died. The findings in Figure 5 show how different diseases impacted mortality predictions over time. Figure 5 shows that 2 *ICD-10* chapters, “diseases of the circulatory system” and “cancer,” had important changes during this period. By associating the findings of step 1 with those of *step 2*, we can begin to understand the factors contributing to decreased mortality over time, but it does not provide the “full picture.”

The DIS semantic characterization step, which measures how the contexts of the independent variables relate to those of the dependent variables over time at a more semantic level*,* yields interesting results that complement the previous ones. Multimedia Appendix 6 shows an example of such a result, that is, changes in similarity for the “dysphagia following stroke” ICD-10 code within the MIMIC-IV data set [18]. There has been an increase in the cooccurrence of many obesity-related ICD codes between the 2011 and 2013 and 2017 and 2019 time slices. This is aligned with general observations of the increase in obesity prevalence in the overall US population. It is worth noting that this technique does not allow us to draw causal conclusions but instead focuses on the correlation and cooccurrence changes. The cooccurrence of death and “cancer,” as well as the presence of “external causes,” has decreased over the period, possibly indicating a reduction in iatrogenic events, improved cancer treatment leading to lower lethality, or that patients with cancer are receiving more end-of-life care at home. This may be an explanation as to why overall in-hospital mortality has decreased in this data set.

As overall mortality decreases, patterns affecting the decrease of similarities between entities, such as the lethality of circulatory diseases, unchanged. This means that increases in similarity with the outcome may be simply due to the decrease in the lethality of other groups. To investigate this, we filtered the data only for cancer disease codes, as in Multimedia Appendix 5. The figure reveals important decreases in mortality in mostly severe and hard-to-treat cancers, such as brain, colon, lung, and secondary (metastatic) tumors.

It is also possible that the observed patterns may be attributed to multiple factors at the same time. For instance, recent policy changes favoring home care for patients who are terminally ill may influence who dies in the hospital. If these patients are more likely to die at home, we might have a “survivorship bias,” where mostly the ones who did not die received hospital care and the patients who were terminally ill were sent back home. Over this time frame, there were important advances in immunobiological therapies for tumors, such as lung cancer, as well as early diagnostic techniques that have made it possible to cure some early cases when the tumor is still resectable. Combining these factors yields a lower lethality, which has decreased over time despite an increase in the total number of patients with neoplasm, as shown in Figure 6.

In summary, the application of the DIS methodology to the MIMIC-IV data set allowed us to determine important trends that help understand certain phenomena observed in the data. Moreover, it facilitates the formulation of interesting hypotheses, which are harder to validate based only on the data themselves. Nevertheless, in a real-world scenario, such hypotheses could be the subject of further investigation using other data sources, such as official policy implementation records, country-wide demographic records, or even published literature.

### Summary of the Main Results of Applying DIS to the Brazilian COVID-19 Registry Data Set

The *drift detection* step, especially using the Jensen-Shannon divergence, revealed important data drifts in the Brazilian COVID-19 Registry data set, which commenced approximately at the same time interval as the vaccination rollout in Brazil, between late 2020 and early 2021 [14]. The initial characterization revealed a trend toward decreasing mortality over time, with the steepest decrease closely matching our drift detection. This means that thus far, there has been an important variable distribution shift as well as a change in the distribution of the outcome itself.

We analyzed how the top 5 highest Pearson correlation variables behaved over time (Figure 8). Figure 8A shows how the relative ranking and correlation of the best predictors of death changed over the course of the pandemic, with features such as “age” being the strongest predictors at the early stages and gradually becoming less predictive over time. Figure 8A also shows how patient severity markers, such as “FiO_2_” and “altered level of consciousness,” gradually became more important predictors over time, hinting at the change from “older patients dying from COVID-19” to “patients who were severely ill at admission dying from COVID-19.” From our analysis, the patient’s age is shown to be a consistently robust predictor of COVID-19–related hospitalization and death. In Figure 8B, we show the median age of the patients who died from COVID-19. This shows how one of the most predictive features in this data set has changed over time, with the median dying age decreasing from approximately 63 years at the peak of the pandemic to approximately55 years in a time frame coinciding with the start of the vaccination campaign in Brazil [5]. However, the median age starts to rise again, possibly relating to another drift, such as the emergence of new viral strains that can disproportionately affect the older adult population. This fluctuation in the median age of deceased patients leads to the aforementioned deterioration of the correlation scores. Furthermore, this pattern with the age variable decreasing over time is consistent with how the vaccines were rolled out to the public, with older age groups being prioritized for vaccination [46]. If these groups received vaccines earlier and consequently reduced their probability of death, this would likely reduce the median and mean deceased patients’ ages.

The main results of the semantic characterization step (Multimedia Appendices 10-12 and Figures 8-10), where we compared the semantic vectors for the “death” outcome in 2020 and 2021, validate several findings from the initial characterization step and introduce new findings. For instance, the results show a decrease in similarity between the outcome and older groups (eg, the age groups “84-105” years vs “62-83” years) with an increase in similarity between the outcome and younger groups. This validates the findings in Figure 8A, where median age declines steadily up until roughly September 2021. Figure 8A also shows how the “death” outcome had an increase in similarity to several disease severity markers, such as lower admission serum sodium, lower admission arterial blood pressure, fewer comorbidities, and lower FiO_2_. This potentially indicates that, when compared to 2020, patients who died in 2021 were more severely ill at admission, had fewer comorbidities, and were younger (presumably unvaccinated). This is a significant pattern change, especially compared to the bulk of deceased patients in the initial chunk, who were mostly older adults with lower severity at admission. This change in pattern implies that, at the analyzed time frame, patients who were young and severely ill at admission were more common among patients who were dying. However, this should be analyzed in conjunction with the previous findings from the other steps. For instance, we know that the overall mortality has decreased, and this patient profile (young and severely ill at admission) could also be present in the first temporal chunk. What possibly happened was the removal of a significant portion of older patients who were dying from the population through events such as vaccination, as evidenced by the reduced mortality and diminished predictive power of age.

To conclude, the DIS analysis hints at the central role of vaccination in the COVID-19 pandemic, which reduced the odds of older patients dying from the disease following the rollout of the vaccines. This hypothesis was raised by the alignment between the detected data drift and mortality reduction during the vaccination period. Additionally, the observed decrease in the median age of the patients who were dying corresponded to the age-stratified vaccination strategy. Furthermore, the shift of mortality burden to patients who were young and severely ill upon admission, who were likely unvaccinated, demonstrates how they possibly kept dying while this process unfolded.

### Limitations

We have proposed a methodology to discover and interpret temporal shifts in health care data. While our approach provides valuable insights by uncovering many correlations and semantic connections, DIS still cannot establish causal relationships outcomes and semantic units. The causal part is only hypothesized and inferred, but the methodology does not go so far as to return causal links for arbitrary outcomes. Furthermore, we have not applied the methodology to certain relevant health care domains, such as images (eg, x-rays, computed tomography, or ultrasound) and wavelets (eg, electrocardiograms or electroencephalograms).

That said, here, we offer some insights into how we could apply DIS to handle temporal shifts in nonquantitative data or raw magnetic resonance imaging data. For this, we would first need to obtain a distributed representation of the data in such a manner that samples from similar patients have similar embedding vectors. For instance, we could use DINOv2 embeddings or contrastive language-image pretraining embeddings in images. This type of pretrained neural network exists for multiple data types, which facilitates its application to multiple domains. From the embeddings, we can apply the first step of our methodology as applied to tabular data, computing Jensen-Shannon divergence (or autoencoder errors, classifier errors, etc) to detect whether a drift exists in the data. Exploring these data in the second step presents some challenges, as it might involve exploring both the embedding and raw data spaces. For instance, we can use clustering and centroid analysis (applied to the embeddings) to find samples where the drift is particularly pronounced. Then, we can go back to the raw data and analyze the samples to check for patterns. In essence, the third step remains similar in nature. The idea is to train a neural network model such that the embeddings of the samples closely resemble the embeddings of the outcomes experienced by those patients over time. One such way to obtain these embeddings, starting from pretrained ones, is to use losses such as the triplet loss to approximate patient sample embeddings from outcome embeddings. The interpretation of the triplet loss, as presented in our paper, will change according to the temporal granularity of the samples. If the data have high temporal granularity, the positive pairs (which the loss will learn to represent more closely in space) will obey an ordered sequence of events. For instance, 2 magnetic resonance imaging tests will be proximate if they belong to the same patient and happen close to each other in time and if they are visually and semantically similar. Conversely, if the data have low temporal granularity, the embeddings should be learned to align patient samples to their outcome embeddings. Then, for the analysis of such embeddings, we would have to analyze the raw data samples closer to the outcome embeddings. If one splits the time, say, in 2 years and is working with the “death” outcome, one would be expected to have 1 such outcome for each year. Then, analyzing the samples closer to each of the outcome embeddings should help build an understanding of the relevant changes in a more generalized setting, and this might require some domain expertise. We intend to explore these ideas in future work.

Finally, we cannot claim that our 3 steps (encompassing the “if,” “what,” and “why” of a data drift) are a comprehensive list of all possible steps to analyze a temporal shift. Instead, we believe our steps to be a minimum required subset. While it is possible that these steps might not cover all possible situations, they allowed us to obtain interesting insights from the 2 data sets presented in our work, as discussed earlier. We and other researchers plan to continue to study, extend, and adapt this methodology in future work to test the limits of our approach and whether new steps or a refinement of the ones proposed at the fiber granularity level is necessary.

We intend to explore methods for enhancing models’ resilience to data drifts, as well as examine different health care–relevant data types, such as images, wavelets, and multimodal data.

### Conclusions

We have proposed DIS, a temporal data drift methodology for analyzing the changes in health outcomes and variables over time while discovering contextual changes for outcomes in large volumes of data. We applied DIS to 2 very different case studies and demonstrated how it can provide valuable insights into changing patterns in the data and the underlying reasons driving such changes.

The DIS methodology goes beyond simple detection; it comprehensively characterizes temporal data drifts. By analyzing the underlying causes, patterns, and magnitudes of drifts, health care stakeholders can gain a deeper understanding of the factors influencing data changes over time. This deeper understanding has practical implications for health care organizations, allowing them to improve patient care, optimize resource allocation, and enhance operational efficiency by leveraging the insights gained from monitoring and characterizing temporal data drifts.

The practical implications of our methodology are far-reaching. Early detection of data drifts can trigger timely interventions, enabling proactive adjustments to treatment plans, health care policies, and quality improvement initiatives. Our methodology empowers health care practitioners and data analysts to effectively monitor and manage temporal data drifts, ultimately leading to better health care outcomes and informed decision-making processes.

## Appendix

Multimedia Appendix 1 Modeling of the centroids as the arithmetic mean of the features in each outcome group. Co is the centroid of cluster O, XCO is the matrix of attributes including all patients in the outcome O, and |CO| is the number of patients in the outcome group O.

Multimedia Appendix 2 The “anchor_year_group” variable on the Medical Information Mart for Intensive Care, version IV (MIMIC-IV) data set. Within each “anchor_year_group,” the actual dates are masked, making it possible to have only a rough estimate of when the patient was at the hospital.

Multimedia Appendix 3 Lethality over time in the Medical Information Mart for Intensive Care, version IV data set.

Multimedia Appendix 4 Drift of the arithmetic mean of each outcome class over time, as measured by cosine distances between each class’s means when compared to the mean of the first “anchor_year_group” in the Medical Information Mart for Intensive Care, version IV data set.

Multimedia Appendix 5 Evaluation of the drivers of lethality data drift in the Medical Information Mart for Intensive Care, version IV data set.

Multimedia Appendix 6 Changes in co-occurrence for the “dysphagia following stroke” International Classification of Diseases in the Medical Information Mart for Intensive Care, version IV data set.

Multimedia Appendix 7 Validation of the data drift in cancer patients. On the left, we show the increase in the absolute number of cancer patients, while on the right, we show the overall lethality reduction for this disease group.

Multimedia Appendix 8 Cluster analysis of the Medical Information Mart for Intensive Care, version IV data set. (A) Top 5 highest-valued features per cluster. (B) Relative frequency of each cluster over time.

Multimedia Appendix 9 Drift of the arithmetic means of the dying patients versus the overall population over time, as measured by cosine distances between each class’s means on each time chunk over time, in the Brazilian COVID-19 Registry data set.

Multimedia Appendix 10 Lethality over time in the Brazilian COVID-19 Registry data set.

Multimedia Appendix 11 Top 15 largest increases and decreases in similarity between the “death” tokens for 2021 and 2020 in the Brazilian COVID-19 Registry data set.

Multimedia Appendix 12 Cluster analysis of the Brazilian COVID-19 Registry data set. (A) Top 5 highest-valued features per cluster. (B) Relative frequency of each cluster over time.

## Abbreviations

DIS: detection, initial characterization, and semantic characterization
FiO2: fraction of inspired oxygen
ICD: International Classification of Diseases
ICU: intensive care unit
KL: Kullback-Leibler
MIMIC-IV: Medical Information Mart for Intensive Care, version IV
ML: machine learning
NLP: natural language processing
PCA: principal component analysis
TF-IDF: term frequency–inverse document frequency
