# Supplementary figures and images for: A New Natural Language Processing–Inspired Methodology (Detection, Initial Characterization, and Semantic Characterization) to Investigate Temporal Shifts (Drifts) in Health Care Data: Quantitative Study
Source: JMIR Med Inform. 2024 Oct 28;12:e54246. doi: 10.2196/54246 (PMC11555458; doi:10.2196/54246)

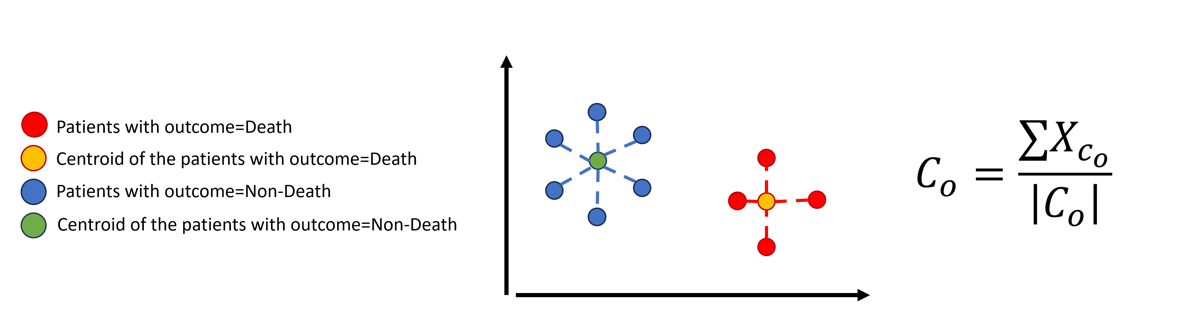

Supplement: Multimedia Appendix 1 [file medinform_v12i1e54246_app1.png]

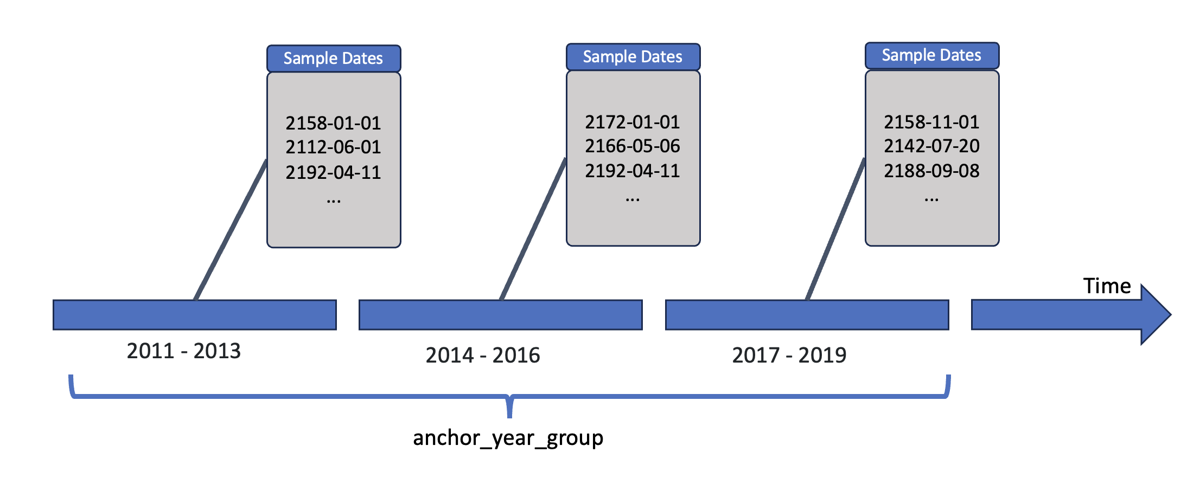

Supplement: Multimedia Appendix 2 [file medinform_v12i1e54246_app2.png]

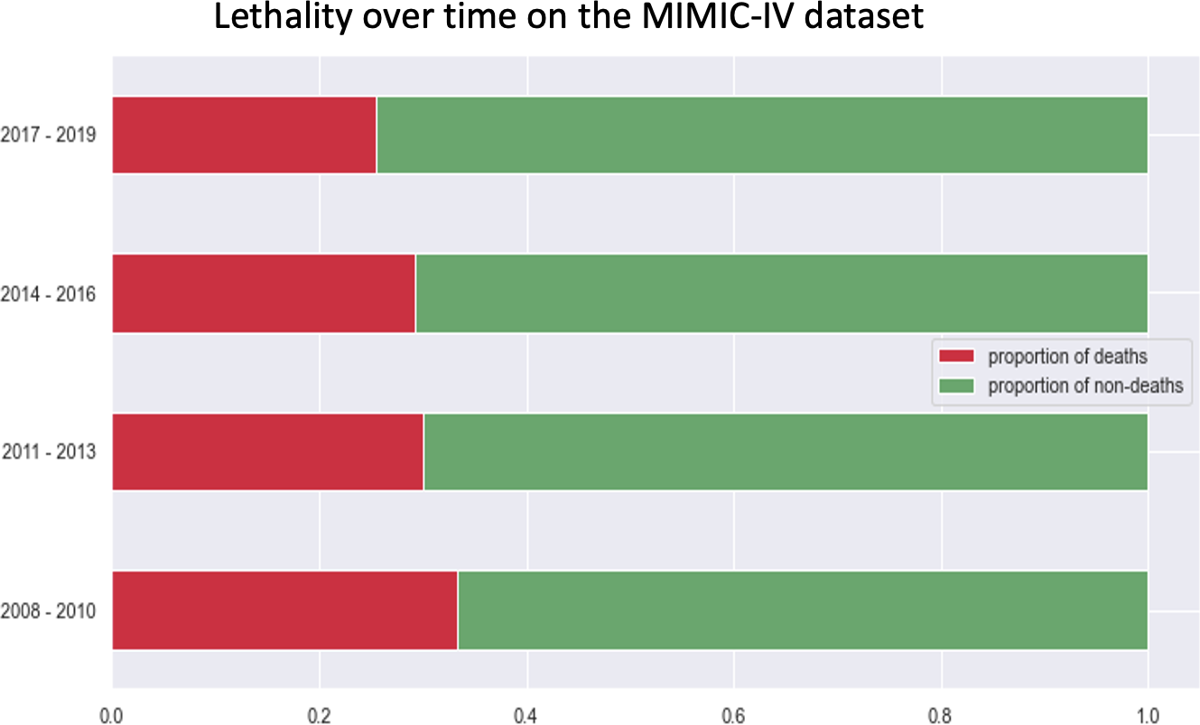

Supplement: Multimedia Appendix 3 [file medinform_v12i1e54246_app3.png]

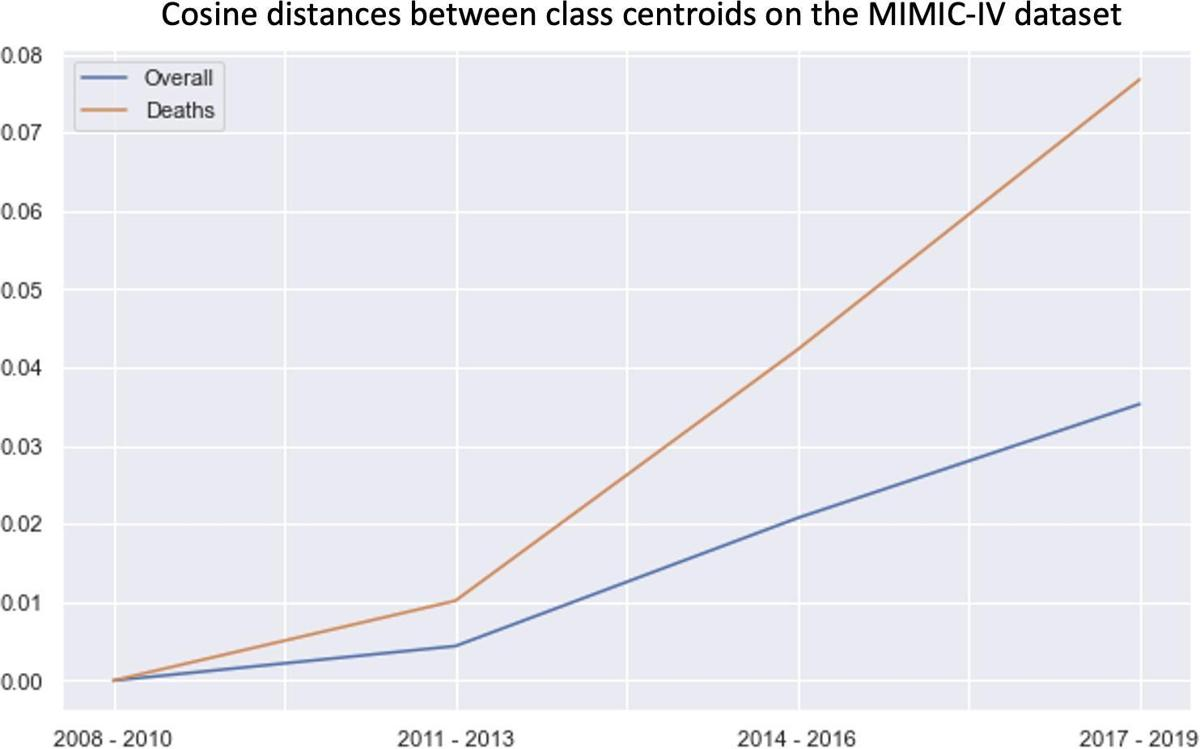

Supplement: Multimedia Appendix 4 [file medinform_v12i1e54246_app4.png]

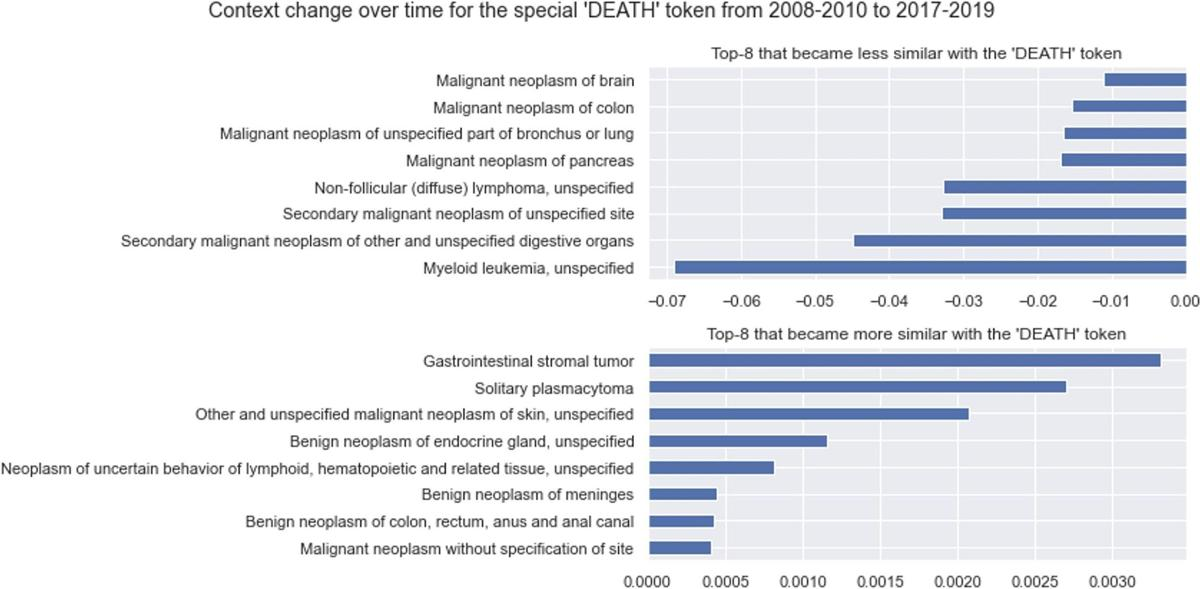

Supplement: Multimedia Appendix 5 [file medinform_v12i1e54246_app5.png]

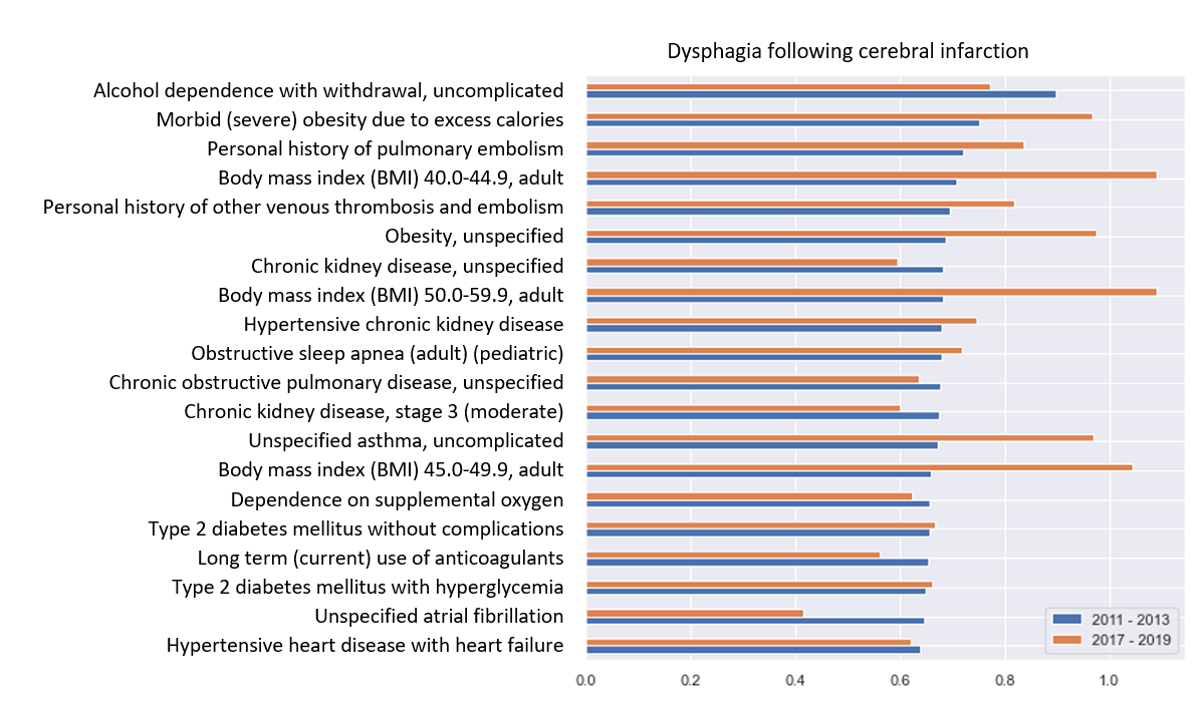

Supplement: Multimedia Appendix 6 [file medinform_v12i1e54246_app6.png]

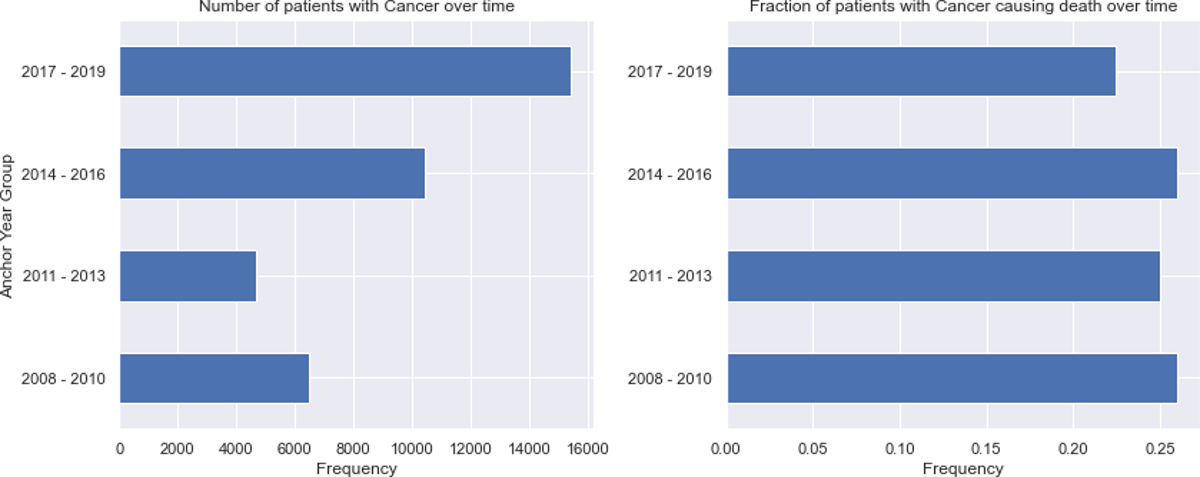

Supplement: Multimedia Appendix 7 [file medinform_v12i1e54246_app7.png]

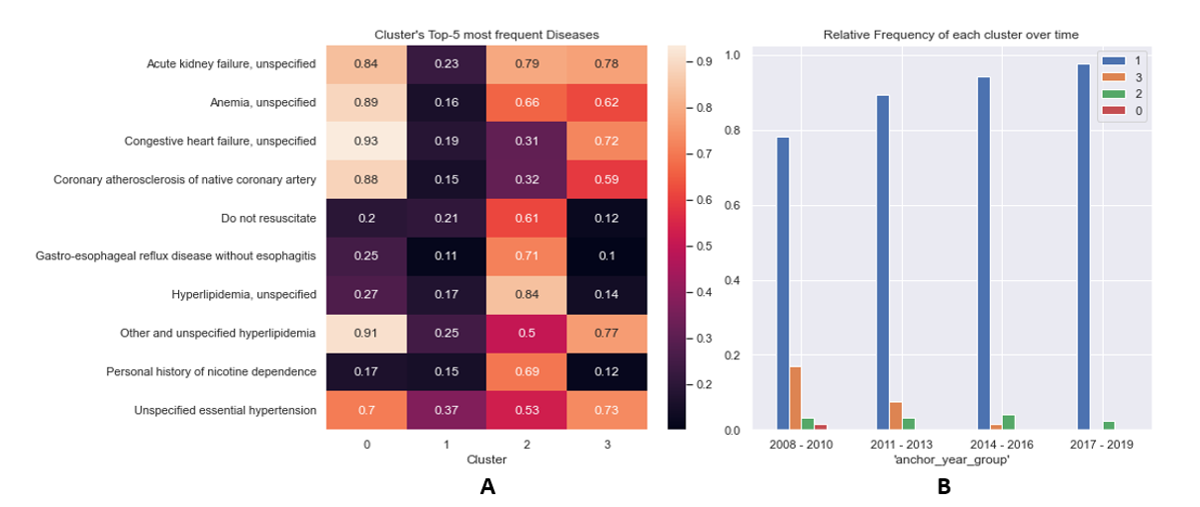

Supplement: Multimedia Appendix 8 [file medinform_v12i1e54246_app8.png]

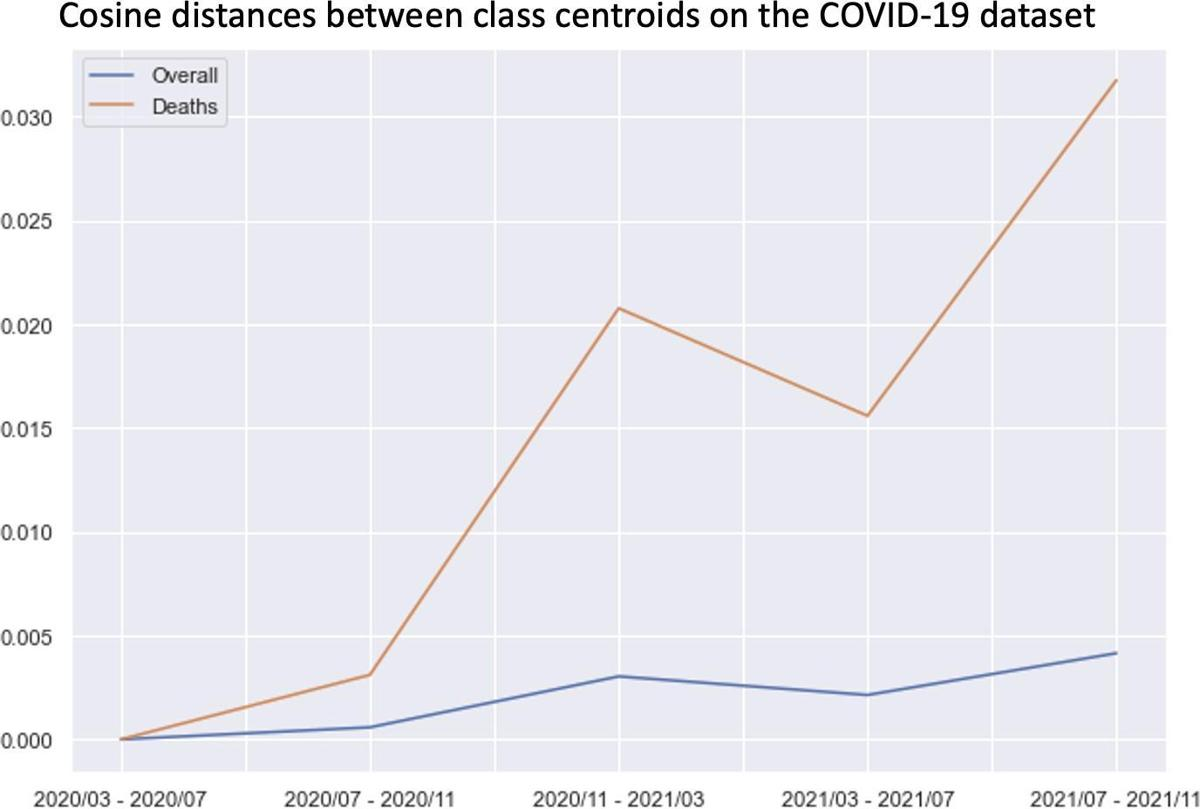

Supplement: Multimedia Appendix 9 [file medinform_v12i1e54246_app9.png]

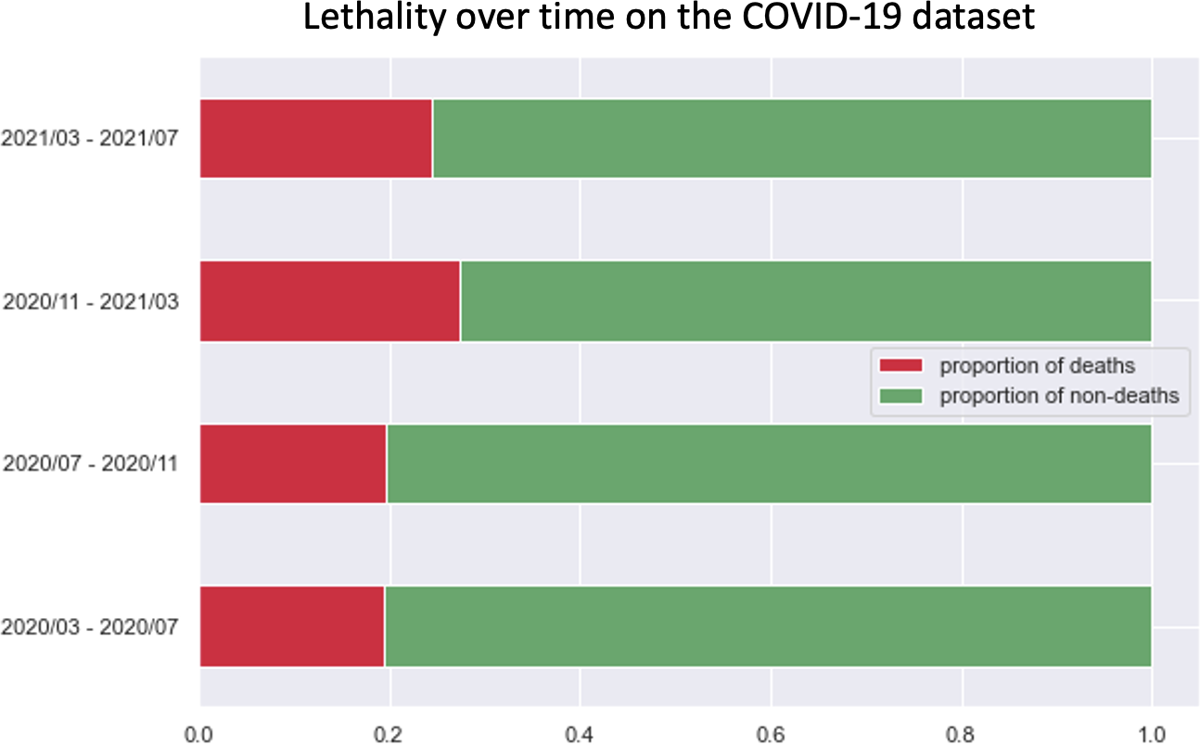

Supplement: Multimedia Appendix 10 [file medinform_v12i1e54246_app10.png]

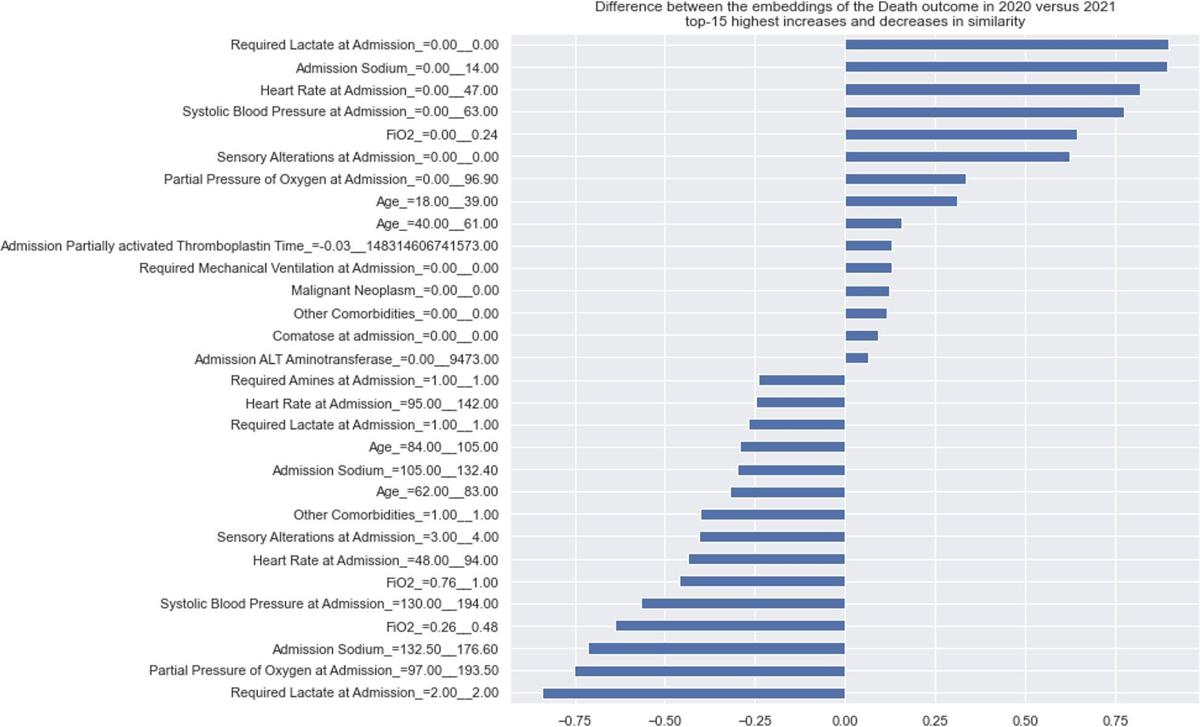

Supplement: Multimedia Appendix 11 [file medinform_v12i1e54246_app11.png]

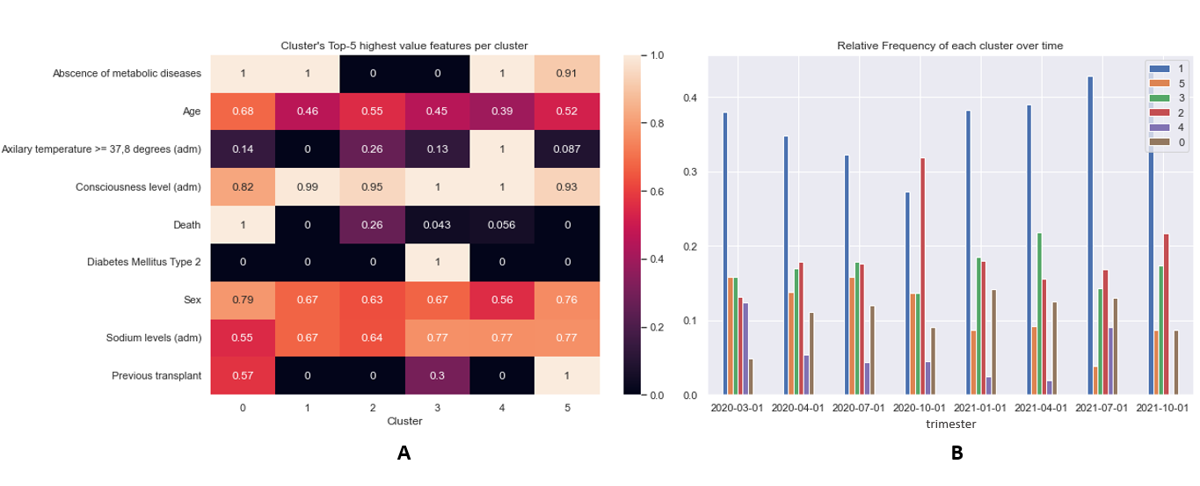

Supplement: Multimedia Appendix 12 [file medinform_v12i1e54246_app12.png]
